# Divergent B-cell and cytotoxic TNK cell activation signatures in HLA-B27-associated ankylosing spondylitis and acute anterior uveitis

**DOI:** 10.3389/fimmu.2025.1546429

**Published:** 2025-03-07

**Authors:** Eisa Mahyari, Sean Davin, Kimberly Ogle, Emma Fale-Olsen, Carley Shaut, Tammy M. Martin, Jasvinder S. Ahuja, Eric Suhler, Atul Deodhar, James T. Rosenbaum, Tejpal Gill

**Affiliations:** ^1^ Oregon National Primate Research Center, Oregon Health and Science University, Beaverton, OR, United States; ^2^ Casey Eye Institute, Oregon Health and Science University, Portland, OR, United States; ^3^ Laboratory of Immunogenetics, Oregon Health & Science University, Portland, OR, United States; ^4^ Division of Arthritis and Rheumatic Diseases, Department of Medicine, Oregon Health & Science University, Portland, OR, United States; ^5^ Legacy Devers Eye Institute, Portland, OR, United States; ^6^ Corvus Pharmaceuticals, Burlingame, CA, United States

**Keywords:** ankylosing spondylitis, acute anterior uveitis, HLA-B27, single cell CITE sequencing, immunophenotype

## Abstract

Ankylosing spondylitis (AS), also known as radiographic axial spondyloarthritis (r-axSpA), is an immune-mediated inflammatory disorder frequently associated with acute anterior uveitis (AAU). Both conditions share a strong association with the genetic risk factor, human leukocyte antigen (HLA)-B27. However, the immunophenotype underlying HLA-B27-associated AS and/or AAU pathophysiology remains known. Using cellular indexing of transcriptomes and epitopes (CITE-Seq) in a well-characterized cohort of 25 subjects—including AS (HLA-B27^pos^), AS+AAU (HLA-B27^pos^), AAU (HLA-B27^pos^), HCs (HLA-B27^pos^), and HCs (HLA-B27^neg^); *N* = 5/group—we identified transcriptomic differences at the single-cell level, along with differentially expressed cell surface markers. Our study elucidates both shared and distinct immune alterations linked to HLA-B27 and disease. Furthermore, we employed sparse decomposition of arrays (SDA) analysis, an unsupervised machine learning method, to examine the high-dimensional transcriptional landscape of our data and identify complex and nonlinear relationships. Our study identified HLA-B27- and disease-specific transcriptomic differences in AS and AAU. The immune profiles of AS+AAU closely resembled those of AS, suggesting AS plays a dominant role in immune dysregulation. SDA analysis further revealed dysregulated B-cell maturation and activation in AS subjects, whereas AAU subjects exhibited an enrichment of cytotoxic effector function in T and NK cells. However, both AS and AAU exhibited myeloid cell activation, a key process in initiating and sustaining inflammation. Additionally, both AS and AAU subjects showed a dampening in homeostatic function, i.e., the balance between identifying and actively eliminating foreign pathogens while preventing an immune response against self-antigens, suggesting that inflammation may arise from immune dysregulation. In conclusion, our results highlight overlapping myeloid effector involvement, along with distinct immunophenotypic responses, such as a decrease in naive B cells in AS subjects and a reduction in the CD8/NK cell population in AAU subjects. These results highlight a distinct set of immune mediators driving AS and AAU pathogenesis. Future studies incorporating HLA-B27-negative AS and AAU patients, along with validation of B-cell and myeloid dysfunction in these diseases, may provide novel biomarkers and therapeutic targets.

## Introduction

Ankylosing spondylitis (AS), or radiographic axial spondyloarthritis (r-axSpA), is a prevalent form of inflammatory arthritis that primarily affects the sacroiliac joints and spinal entheses (1). It is characterized by chronic inflammation, leading to extensive new bone formation and vertebral fusion, which cause pain, reduced mobility, and disability (2). Beyond musculoskeletal involvement, AS often presents with systemic manifestations, including inflammation of the gut, skin, and eyes, contributing to significant morbidity and disease burden (3–6). AS frequently overlaps with acute anterior uveitis (AAU), a condition characterized by painful red eyes, photophobia, and, in some cases, decreased visual acuity (7). AAU is the most common extra-articular manifestation of AS, affecting 20%–50% of AS subjects (7). Epidemiological data suggest a rising incidence of AS and associated AAU over the past two decades (8). Therefore, accurate diagnosis and clinical stratification are crucial for improving patient outcomes.

Subjects with AS and AAU share a strong genetic risk factor: the human leukocyte antigen HLA-B27 (9, 10). While approximately 90% of AS subjects and 60% of AAU subjects carry the HLA-B27 allele, it is also present in 4%–7% of healthy individuals (11). HLA-B27 enhances immune protection against various viral pathogens, including HIV (12) and influenza (13), by restricting viral epitopes and expanding its role in antigen presentation—both to NK cells via KIRDL1 receptors (14, 15) and to T cells through atypical pathways (16). However, despites its antiviral protective effects, gene expression analysis of peripheral blood mononuclear cells (PBMCs) from AS (17, 18) and AAU (19, 20) subjects, as well as gut tissues from HLA-B27 transgenic rats, has revealed a dysregulated immune response characterized by an increased inflammatory signature driven by IL-17/23, TNF, and IFN-g (21). In addition, HLA-B27 has been implicated in gut microbial dysbiosis observed in AS (22, 23), AAU (24, 25), and HLA-B27^pos^ healthy controls (HC) (26). Thus, HLA-B27 simultaneously enhances antiviral immunity, alters gut microbe–host immune response interactions, and predisposes carriers to the development of AS, AAU, and other spondyloarthritis.

To address the complexity of immune responses in AS and AAU, it is crucial to analyze immune cells at a resolution that captures both cellular diversity and functional states. Recent single-cell studies of PBMCs from AS subjects have revealed altered immunophenotypes compared to healthy controls (HCs) (27–29). Despite the high co-occurrence of AS with AAU (30), single-cell immunophenotyping of AAU subjects, both with and without AS, remains unexplored. Additionally, the effect of the (HLA)-B27 allele on the immunophenotype in HCs also unknown. While both AS and AAU are associated with HLA-B27, they may involve distinct immune mechanisms, reflecting differences in their underlying pathophysiology. Identifying the specific immunophenotypes responsible for AS, AAU, or their coexistence (AS+AAU) is therefore essential. In this study, we employed cellular indexing of transcriptomes and epitopes by sequencing (CITE-Seq) to investigate transcriptomic differences at the single-cell level and assess differentially expressed cell surface markers in HLA-B27^pos^ AS, AAU, and AS+AAU subjects. To understand the effects of the disease and the HLA-B27 allele, we compared these groups to HCs with and without HLA-B27.

## Material and methods

### Study design and participants

We conducted a prospective cohort study at Oregon Health and Science University (OHSU), approved by the OHSU Institutional Review Board. Written informed consent was obtained from all participants. Subjects diagnosed with AS, AS+AAU, and AAU alone were recruited from OHSU rheumatology and uveitis clinics (subject characteristics detailed in **Supplementary Table S1**). AS diagnoses, with and without AAU, were confirmed by a rheumatologist (JTR/AD) based on the modified New York (NY) criteria (31). Given the limited cohort size, we focused on HLA-B27:05 (referred to as HLA-B27) subjects with well-characterized disease and radiographic evidence of bilateral sacroiliitis. AAU diagnoses were confirmed by an ophthalmologist (ES) at OHSU. Subjects were excluded if they were under 18 years old or pregnant. Current medications, including biologics and nonsteroidal anti-inflammatory drugs (NSAIDs), were documented. To enhance cohort uniformity, AS and AAU subjects with psoriasis, peripheral arthritis, or inflammatory bowel disease (IBD) were also excluded. The Bath Ankylosing Spondylitis Disease Activity Index (BASDAI) was administered to AS, AS+AAU, AAU, and HC groups. HCs were recruited via flyers posted throughout OHSU, with both HLA-B27^+^ and HLA-B27^−^ individuals included (*N* = 5/category). All subjects provided blood samples for PBMC isolation and genomic DNA extraction for HLA-B typing. Genomic DNA was extracted and analyzed using the LABType XR HLA-B SSO typing kit from One Lambda (Thermo Fisher Scientific, Waltham, MA, USA; RSSOX1B) as described in our previous study (23). HLA-B27 allele information, following current standard nomenclature (32), for all HLA-B27 positive and negative subjects is detailed in **Supplementary Table S2**.

### PBMC isolation, cryopreservation, and thawing

PBMCs were isolated from collected blood samples using Ficoll–Paque PLUS density gradient centrifugation (GE Healthcare, CA, USA) following the manufacturer’s instructions. Briefly, blood was layered over Ficoll–Paque media and centrifuged to separate the PBMC layer, which was then resuspended and washed in 40 ml of 1× PBS. The isolated PBMCs were counted using a TC20 Automated Cell Counter (Bio-Rad Laboratories, Hercules, CA, USA), and cell viability was assessed using trypan blue exclusion, with a viability threshold of > 90% required for further analyses. PBMCs were frozen using a resuspension buffer and two times freezing media, dispensed into prechilled cryovials and stored at − 80 C for 4–24 h before being transferred to liquid nitrogen for long-term storage. Thawing was performed according to the 10× Genomics, Pleasanton, CA, USA Single-Cell Protocol (Fresh Frozen Human PBMCs for Single-Cell RNA Sequencing, CG00039), which outlines cryopreservation and thawing of human PBMCs. Cryovials were removed from liquid nitrogen and immediately thawed in a 37°C water bath for 2–3 min. In a biosafety cabinet, thawed cells were transferred to a 50-ml conical tube, and 1 ml of warm complete growth medium was added dropwise with gentle shaking. The cells were serially diluted five times (1:1), with a 1-min wait between each addition, followed by centrifugation at 300 rcf for 5 min. The supernatant was removed, leaving 1 ml of media, to which 9 ml of complete growth media was added. Cell concentration was assessed using a hemocytometer with trypan blue staining and an automated cell counter. Two million cells were transferred into a new 50- ml tube, and 1 ml of 1× PBS with 0.04% BSA was added five times (1 ml at a time), followed by centrifugation at 300 rcf for 5 min. Assuming a 50% loss, cells were resuspended at 12,000 cells/µl and processed for cell surface staining.

### Cell surface staining

Cell surface proteins were labeled using BioLegend, San Diego, CA, USA TotalSeq™-B following the 10× Genomics Cell Surface Protein Labeling for Single-Cell RNA Sequencing Protocols with Feature Barcode technology (CG000149). This protocol details antibody-oligonucleotide conjugation and cell surface protein labeling for use in single-cell RNA sequencing with Feature Barcode technology. Thawed PBMCs were resuspended in 50 µl of chilled 1× PBS and 4% BSA, and 5 µl of Human TruStain FcX was added, followed by a 10-min incubation at 4°C. The antibody mix supernatant was then added, and the cells were incubated for 30 min at 4°C. Thorough washing postincubation was critical to obtaining high-quality data. Cells were washed with 3.5 ml of chilled BSA with 0.04% BSA and centrifuged at 300 rcf for 5 min. The supernatant was removed, and the pellet was resuspended in 100 µl of room-temperature PBS. This wash-step was repeated three times, after which the cells were immediately processed for 10× Genomics Single-Cell protocols.

### Single-cell CITE sequencing

Single-cell transcriptomics (scRNA-Seq) enables detailed analysis of individual cell gene expression, revealing the heterogeneity within immune cell populations and how HLA-B27 expression might vary across different cell types.

#### Single-cell preparation and library construction

Single-cell suspensions for scRNA-Seq were prepared using the Chromium Single Cell 3′ Library and Gel Bead Kit v3 (10× Genomics), following the manufacturer’s protocol. This process encapsulates individual cells into droplets with barcoded beads for reverse transcription, generating cDNA from mRNA transcripts present in each cell. The prepared libraries were sequenced on an Illumina, San Diego, CA, USA NovaSeq platform, targeting a depth of approximately 50,000 reads per cell.

### Computational and bioinformatics analysis of CITE-Seq data

#### Count data derivation

Sequencing data per sample were aligned to the human reference genome Human_GRCh38.p14_Ensembl (GCA_000001405.29) using the *Homo_sapiens*.GRCh38.110 genome annotation files (gene model) with Cell Ranger 6.1.1. These individual count matrices were pooled to construct a comprehensive dataset encompassing all samples under study.

#### Cellular quality control

Initial quality control (QC) and preprocessing of scRNA-Seq data were performed using the Seurat (33) (v5) R package. Cells with fewer than 200 or more than 5,000 detected genes, and more than 20,000 UMIs, were excluded to remove empty droplets or dying cells. Data were further filtered to exclude cells with high mitochondrial gene content (> 15%), indicative of apoptotic or damaged cells. Gene expression (GEX) and protein expression (CITE) matrices from each run were merged, retaining only cells with both for downstream analysis. Lastly, all cells across all samples were pooled to create a final combined object.

#### Data processing

The combined dataset was normalized using the “LogNormalize” method in Seurat. Three thousand highly variable genes were identified using the “FindVariableFeatures” function in Seurat, which were then used as input to reduce the dimensionality of the data prior to principal component analysis (PCA). The ElbowPlot function was used to determine the number of principal components (15) to include in clustering analyses.

#### Unsupervised clustering

Unsupervised graph-based k-nearest neighbors clustering, using the original Louvain algorithm implemented in Seurat, was used. The dataset was split by major cell types (i.e., T and natural killer (TNK) cells, B cells, and myeloid cells) to refine and focus the downstream analysis.

#### Classification of major cell-type and subphenotypes

Using canonical markers (protein and RNA), including CD3, CD20, CD14, and C1QA, we define broad labels to subset the combo object into TNK, B, and myeloid cell objects. Each was subsequently reprocessed, including normalization, scaling, feature selection, dimensionality reduction, and clustering. This process enabled the further identification and removal of unexpected doublet clusters. For example, in the B-cell reprocessed data, where all cells effectively expressed the B-cell marker CD20, there were distinctly separated clusters that also expressed CD3 and/or CD14.

To maintain consistency with previous studies that have used Azimuth (33) to identify immunophenotypes in human PBMC samples, we used the level 2 (L2) calls from Azimuth as the basis of our subphenotypes of interest. We then employed our protein-RNA-based call of major cell types to refine the final results, removing unexpected doublet clusters that were excluded from downstream analysis. We confirmed, using protein and RNA markers (**Supplementary Figure S2**), that our calls align with the expected expression patterns described in classical immunological literature.

#### Contrast differential expression analysis

Differential expression (DE) analysis was conducted across multiple comparisons. First, to determine the contribution of HLA-B27 expression to the PBMC immune landscape, we performed DE analysis by contrasting healthy controls (HC) who were HLA-B27^neg^ with HLA-B27^pos^ individuals. Initially, comparisons within major cell types (i.e., bulk analysis of all cells within each major cell type) yielded very few DE genes. To maximize the power of scRNA-Seq, we repeated the analysis at the subphenotype level for each major cell type (Azimuth L2 phenotypes) (34). Next, to assess the contribution of AS, we performed DE analysis by contrasting AS (HLA-B27^pos^) subjects with HC (both with and without HLA-B27), effectively combining the two directed contrasts. Similarly, to evaluate the contribution of AAU, we compared AAU subjects with HC (both with and without HLA-B27). Lastly, to examine subjects with both AS and AAU, we compared this (AS+AAU) group with those having either AS or AAU alone. As before, these comparisons were initially conducted at the bulk level for each major cell type, yielding very few, if any, DE genes. Given the cellular heterogeneity, overlapping transcriptional programs, and immune signatures, DE analysis at the subphenotype level was chosen as the final resolution for reporting DE genes. For all contrasts, the “FindMarkers” function in Seurat (33) was used (with common settings: min.pct = 0.5, min.diff.pct = 0.1, and logfc.threshold = 0.1). A false discovery rate (FDR) correction (35) was applied for multiple testing, with an adjusted *p*-value of < 0.05 considered significant.

### Sparse decomposition of arrays analysis

To extend beyond standard scRNA-Seq analysis—such as cell type enumeration, differential expression analysis, and gene set enrichment—an unsupervised machine learning approach was employed to explore the high-dimensional transcriptional landscape of our data and identify complex, nonlinear relationships. Previous studies with similar analytical needs have successfully utilized sparse decomposition of arrays (SDA) (36) to uncover biological and pathological signatures (37) using scRNA-Seq data. SDA is a soft-clustering approach that decomposes a high-dimensional cells-by-genes expression matrix into two matrices: scores (cells by components) and loadings (genes by components). Several features make SDA advantageous over similar algorithms, such as PCA. Unlike PCA, SDA components are not constrained by orthogonality or variance reduction. In the loadings matrix, each SDA component consists of gene loadings that assign weights to genes, centered around zero. Notably, only a small subset of genes with high absolute magnitudes—either positively or negatively—serve as drivers for each component, enhancing interpretability and biological relevance.

For this study, we ran several SDA runs with an increasing number of components (e.g., 50, 100, 150) on the full PBMC dataset to ensure a diverse set of components was captured. QC analysis of the components was performed to remove aberrant scoring or noisy (e.g., batch effects) components. The remaining components were then deeply examined relative to cell types, subjects, diseases, and other metadata. By integrating the knowledge of the top positively and negatively loaded genes with Gene Ontology (GO) (38) enrichment analysis, several clinically relevant components were identified. Additional components provide insights into PBMC gene expression and cell-type identification but were not associated with pathology, such as HLA-B27, AS, or AAU, and are therefore not discussed in detail.

### Pathway and functional enrichment analysis

We utilized the enrichGO function from the clusterProfiler R package (39) to perform GO enrichment analysis on the top-loaded genes identified through SDA factor analysis. Each SDA component includes all the genes in the dataset, weighted (loadings) differently by their loadings; therefore, the top 100 genes in each direction were selected to provide a functional overview of the biological mechanisms captured by each component. The analysis was conducted across biological process (BP), molecular function (MF), and cellular component (CC) ontologies, with annotations sourced from the org.Hs.eg.db database (for human data). Genes were mapped to GO terms based on their Entrez IDs, and enrichment was assessed using the hypergeometric test. Multiple testing correction was applied using the Benjamini–Hochberg method, with a FDR threshold of 0.05 to determine significant GO terms. While this approach provided a broad functional overview of the biological processes associated with each component, it is inherently constrained by the scope and annotation biases of existing databases, particularly regarding specific tissues and conditions of interest. To evaluate other gene sets, such as the DE genes discussed, we used ShinyGO (40) as a companion tool to our SDA-GO enrichment approach.

### Statistical methods

Differential expression analysis was performed for all described contrasts using the “FindMarkers” function in Seurat (33), with the following parameter settings: min.pct = 0.5, min.diff.pct = 0.1, and logfc.threshold = 0.1. These criteria ensured that only genes expressed in at least 50% of cells in at least one of the compared groups, with a minimum expression prevalence difference of 10%, were included in the analysis. A log2 fold change threshold of 0.1 was chosen to capture subtle but potentially biologically meaningful changes. Given the dataset’s subdivision into multiple immune cell subphenotypes, applying a higher threshold risked overlooking modest yet potentially meaningful differences. To correct for multiple testing, the FDR was calculated using the Benjamini–Hochberg method (35), with an adjusted *p*-value (FDR) < 0.05 considered statistically significant. For functional gene enrichment analysis of SDA components, the top 100 genes with the highest positive and negative loadings from each SDA component were analyzed using the enrichR library (v3.3, ). GO terms and pathway annotations were evaluated, with a FDR-adjusted *p*-value threshold of < 0.05 applied to identify significantly enriched biological processes, ensuring robust identification of functional signatures.

### Validation dataset GSE194315

A previous study (27, 41) on PBMC scRNA-Seq, which included data from AS, HC, and psoriatic arthritis (PsA) samples (GSE194315, https://www.ncbi.nlm.nih.gov/geo/query/acc.cgi?acc=GSE194315), provided processed data and cell type labels. Similar to our study, this dataset also used Azimuth (33) for cell type annotation, making it suitable for validation. We reprocessed these data from raw counts identical to our data, including splitting them into major lineages (TNK, B, and myeloid cells). Next, we projected our SDA model, trained on our dataset (computed as the dot product of the gene loadings matrix and the inverse gene expression matrix), onto the reprocessed GSE194315 data as a Seurat object, using our custom code library functions from the “scCustFx” R package (https://github.com/eisascience/scCustFx).

### User manual for BASSAA v0.4

#### Introduction

The HLA-B27 Ankylosing Spondylitis and Acute Anterior Uveitis Atlas (BASSAA) (currently in Beta v0.4) is a web application built on the Shiny platform to visualize single-cell RNA/CITE sequencing data from our study. This tool allows users to interactively explore the dataset. To enhance usability, we have also incorporated PBMC data from AS and HC donors (GSE194315) alongside our own dataset, providing validation for our SDA-derived results. The primary objective of BASSAA is to facilitate the exploration of single-cell data, allowing researchers to interact with our results and further investigate complex datasets to generate or test new hypotheses.

#### Getting started: introducing the GUI

The “Home Page” serves as the landing tab, providing a summary of how to use and interact with the Shiny App. The “Main Tab” contains the primary selection tools and visualizations.

#### Visualization input panel

The visualization input panel determines the starting point of your analysis. Here, you choose how your data are visualized and categorized:

1. Data origin.

In the current version, we have integrated a UMAP, which can be selected to show cells from either our data, GSE194315, or both. This is the same UMAP shown in the **Supplementary Figures** of our manuscript.

2. Metadata selection.

This set of selection buttons enables the visualizations to focus on/split by specific categories, such as major cell types or experimental conditions.

#### Cell selection panel

Analysis can be tailored by filtering the data based on experimental conditions or cell types.

1. Condition.

One may choose to focus on a specific condition (e.g., “HC only” for healthy controls) or analyze the entire dataset.

2. Major cell type.

The analysis can concentrate on specific cell types, such as T cells (TNKs) or B cells.

#### SDA inputs panel

This panel highlights the SDA components, each representing the underlying signals derived from scRNA-seq data. Users can make their selections through a dropdown menu. The components detailed in the manuscript are presently included in the current version, with plans to expand and add more components in future releases SDA Component Search: Input a numerical component ID to analyze specific biological patterns.

##### SDA score threshold

Scores are filtered to highlight significant patterns in the data, with adjustments to this value changing the sensitivity of visualizations and gene lists.

##### Gene output controls

The number of genes is specified to highlight the top positively and negatively loaded genes. Buttons are available to copy or download lists of these genes, though this feature currently works only locally and not on the website.

#### Example to get an SDA component from a gene

To identify the gene coexpression networks and their associated roles, enter the gene name in the “Gene Inputs” > “Gene search (text inputs)” section. For example, by entering the gene name “TNFRSF13B” and checking the plot named “Gene Expression (2D)”, the SDA components related to this gene will be displayed. We observe that “TNFRSF13B” appears in the B-cell defect signature (SDA 76), which is the first component (in order of significance for the queried gene, e.g., TNFRSF13B in this case). By entering 76 in the “SDA component search (numerical)” section, we can generate data for the coregulated genes, their pathways, enrichment scores, and additional related information.

SDA components can also be reconstructed using their numbers in the “SDA component search (numerical)” section, independent of the gene input.

## Results

### Clinical characteristics of AS and AAU subjects

To determine the immunophenotypes from AS, AS+AAU, and AAU subjects compared to HC, we performed single-cell CITE seq on PBMCs (N=5/condition) as detailed in **Supplementary Methods**. To dissect the effect of disease status and host genetics (HLA-B27), AS and AAU subjects were compared with HC with and without HLA-B27. Since AS and AAU are frequently concomitant diseases that overlap in subjects, we included AS+AAU subjects to determine their overlapping effect on the immunophenotype. We employed a well-characterized cohort with all AS subjects having radiographic evidence of bilateral sacroiliitis and AAU subjects having active eye inflammation at the time of blood collection. The AS, AAU, and AS+AAU subjects were age, sex, and body mass index (BMI) matched with all HC (with and without HLA-B27), except for one comparison in which AS subjects had significantly higher BMI compared to HLA-B27^pos^ HC (*p* = 0.037). AS subjects with AAU had a significantly (*p* = 0.0044) increased BASDAI than HC without HLA-B27 (**Supplementary Table S1**).

### Phenotyping and quantifying major cell-type lineages

Single-cell CITE-Seq of PBMCs from 25 subjects yielded 408,982 cells in total. Following cellular quality control, 86.5% of these cells were kept, including 245,206 TNK cells, 22,893 B cells, and 85,776 myeloid cells, which were assigned to 19, 4, and 8 transcriptional subgroups, respectively (**Supplementary Table S3**; **Supplementary Figures S1A–C**). Each of these datasets is represented by a specific transcriptional landscape consisting of protein-coding, long noncoding (lnc) RNA genes, and pseudogenes as shown in previous studies (42–44). Our dataset included a total of 22,846 protein-coding genes, with 20,349 genes in TNK cells, 18,923 genes in B cells, and 20,194 protein-coding genes in the myeloid cell subset. Additional gene types, such as lnc RNA genes and pseudogenes, are detailed in **Supplementary Table S4**. To increase reproducibility in cell-type labeling and align our cell-type labels with those from a previous publication (27), we used Azimuth (33), which identified 17 distinct phenotypes of TNK cells (including CD4 CTL, CD4 naive, CD4 proliferating, CD4 circulating memory T cells [TCM], CD4 effector memory T cells [TEM], CD8 naive, CD8 proliferating, CD8 TCM, CD8 TEM, double-negative [dn]T cells, gamma delta [gd]T cells, innate lymphoid cells [ILCs], mucosal-associated invariant T [MAIT], natural killer [NK] cells, NK proliferating, NK_CD56^bright^, regulatory T cell [Treg]; **Supplementary Figure S1A**). In B cells, four phenotypes were identified (B_inter_, B_mem_, B_naïve_, and plasmablasts; **Supplementary Figure S1B**), and seven phenotypes were identified in myeloid cells (AXL+SIGLEC6+ dendritic cells [ASDCs], CD14+ monocytic cells [CD14 Mono], CD16^+^ monocytic cells [CD16 Mono], type-1 conventional dendritic cells [cDC1], type-2 conventional dendritic cells [cDC2], hematopoietic stem and progenitor cells [HSPC], and plasmacytoid dendritic cell [pDC]; **Supplementary Figure S1C**). To verify these cell type labels, we examined the expression of key cell surface proteins expressed on these cells (**Supplementary Figures S2A–C**) and their paired RNA markers (**Supplementary Figures S2D–F**) for TNK, B, and myeloid cells. These three major cell-type lineages define the focus of this manuscript, as other cell types are either considered debris (e.g., erythrocytes) or are rarely detected and not included in the downstream analysis.

### Distinct immune cell reprogramming toward inflammatory status in HLA-B27-positive HCs

**Figure 1:**
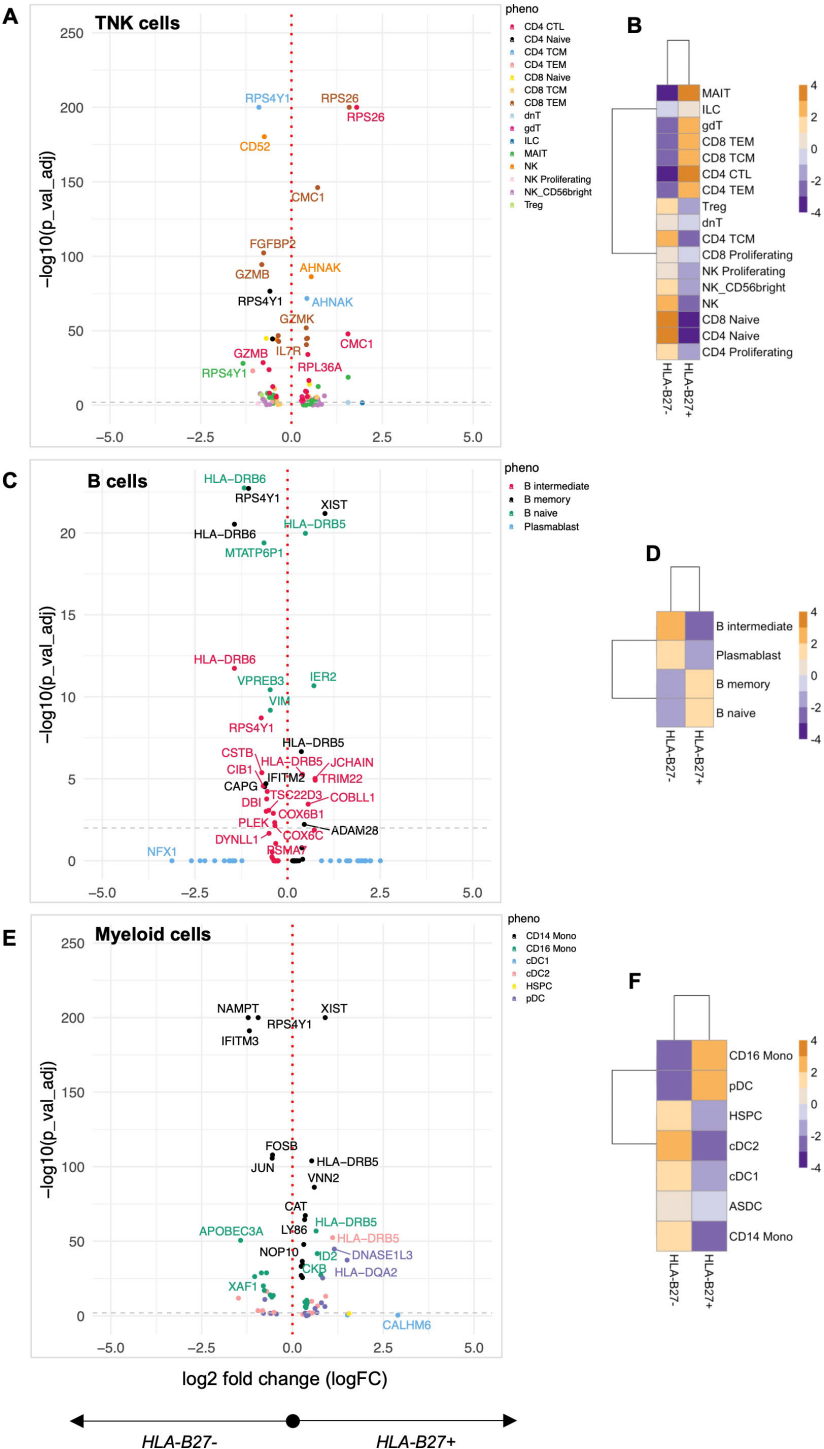
Quantification of the HLA-B27-associated immune landscape in HC. Healthy controls (HC) with and without HLA-B27 (*N* = 5 per group) were compared to identify differential immune features. Differential expression analysis was performed for each of the major cell lineages: **(A)** TNK cells, **(C)** B cells, and **(E)** myeloid cells. Volcano plots illustrate genes with significant differential expression (adjusted *p*-value < 0.05), with uniquely labeled genes representing those with the largest log2 fold changes within each subphenotype. Cellular enrichment for subpopulations within **(B)** TNK cells, **(D)** B cells, and **(F)** myeloid cells was assessed using Chi-squared residuals calculated from observed vs. expected frequencies. The values displayed in the heatmaps represent Chi-squared residuals of observed vs. expected frequencies for each cell subset under the indicated conditions. Positive residuals indicate a higher-than-expected frequency (enrichment), while negative residuals indicate a lower-than-expected frequency (depletion). Heatmaps display the enrichment patterns, with orange indicating enrichment (higher-than-expected frequencies) and blue indicating depletion (lower-than-expected frequencies). These visualizations summarize significant differences in cellular composition between HLA-B27^pos^ and HLA-B27^neg^ HC groups.

To investigate whether HLA-B27 expression impacts the immunophenotype, we compared the transcriptome and cell surface epitopes of specific immune cell subsets in HC with and without HLA-B27. Within HC, we had 82,233 cells in the TNK fraction, 6,189 B cells, and 26,279 myeloid cells, with almost equal representation of distinct cellular populations from HC with and without HLA-B27 (46.85% TNK cells, 53.87% B cells, and 43.22% myeloid cells expressed HLA-B27, **Supplementary Table S3**). DE analysis of these groups showed multiple genes with significantly increased or decreased gene expression (FDR adjusted *p*-value < 0.05) in HLA-B27^pos^ HC in comparison with HLA-B27^neg^ HC (**Figure 1**). Examining all TNK sub-phenotypes, 66 unique genes were identified to differentiate HLA-B27^pos^ in comparison with HLA-B27^neg^ HC (**Figure 1A**). An overall comparison of TNK cell numbers between HLA-B27^pos^ and HLA-B27^neg^ HC is shown as a heatmap with increased numbers shown in orange and decreased cell counts in blue. In context with cellularity, HLA-B27^pos^ HC was enriched for cytotoxic and effector T cells, including MAIT and gdT cells, and had depleted levels of naive CD4/CD8 T cells compared to HLA-B27^neg^ HC (**Figure 1B**). Similarly, in B cells we identified 31 unique DE genes (**Figure 1C**). From a cellular context, HLA-B27^pos^ individuals have higher levels of naive and memory B cells and lower levels of intermediate B cells and plasmablasts compared to the HLA-B27^neg^ individuals (**Figure 1D**). Additionally, in myeloid sub-phenotypes, we identified 58 unique genes (**Figure 1E**; **Supplementary Table S5**); along with increased cell numbers in CD16^+^ monocytes and pDCs in HLA-B27^pos^ HC than HLA-B27^neg^ HC (**Figure 1F**).

Pathway analysis of DE genes was performed using Shiny GO (40), revealing coordinated immune reprogramming across multiple cellular compartments. In TNK cells, we identified a distinct cytotoxicity signature characterized by dysregulation of cytolytic enzymes (*GZMK* up, *GZMB2*, and *FGFBP2* down), coinciding with the observed enrichment of cytotoxic effector populations. This enhanced cytotoxic profile was accompanied by upregulation of *IL7R*, suggesting altered T-cell homeostasis. The myeloid compartment showed activation of key inflammatory regulators, with upregulation of AP-1 transcription factor components (*FOSB, JUN*) and cytokine signaling molecules (*IL6ST*), alongside increased interferon-responsive gene expression (*GBP4*). These changes occurred parallel with expanded CD16^+^ monocyte and pDC populations, suggesting enhanced innate immune surveillance in HLA-B27^pos^ individuals. Notably, we observed changes in cellular stress response pathways across all major immune populations. Differential expression of ribosomal proteins (*RPS26*, *RPL36A*) and mitochondrial factors (*CMC1*) suggests altered protein processing, a finding particularly relevant given HLA-B27’s known propensity for misfolding (45). In B cells, the combination of *HLA-DRB6* modulation and shifts in naive/memory versus intermediate/plasmablast populations indicates altered B-cell maturation and antigen presentation capacity. Together, these pathway-level changes reveal that HLA-B27 expression fundamentally alters immune cell programming HC, either directly or by altering the gut microbiome (26), which could change cell activation/immune status, thus establishing a distinctive immunological baseline that may influence susceptibility to AS and AAU.

### AS subjects exhibit altered TNK, B cell, and myeloid profiles in comparison to HC independent of HLA-B27 status

**Figure 2:**
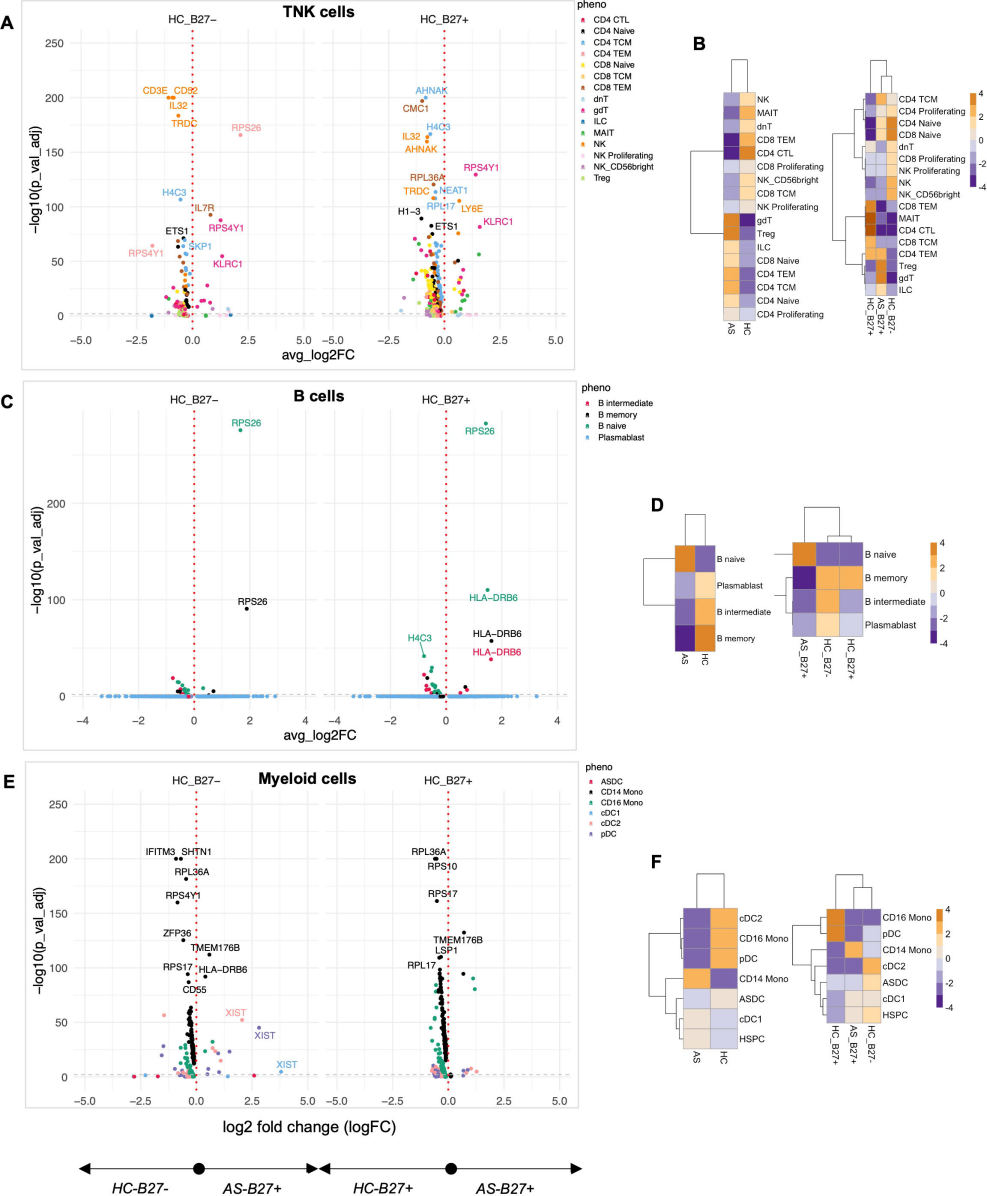
Quantification of the immune landscape in AS. Subjects with ankylosing spondylitis (AS) were compared to healthy controls (HC) with and without HLA-B27. Differential expression analysis was performed for each major cell lineage: **(A)** TNK cells, **(C)** B cells, and **(E)** myeloid cells. Volcano plots display genes with significant differential expression (FDR-adjusted *p*-value < 0.05). Comparisons to HLA-B27^ne^g HC are shown on the left, while comparisons to HLA-B27-positive HC are shown on the right. Genes with the largest log2 fold changes are uniquely labeled in the plots for each subphenotype. Cellular enrichment for subpopulations within **(B)** TNK cells, **(D)** B cells, and **(F)** myeloid cells was assessed using Chi-squared residuals, calculated from observed vs. expected frequencies. The values displayed in the heatmaps represent Chi-squared residuals of observed vs. expected frequencies for each cell subset under the indicated conditions. Positive residuals indicate a higher-than-expected frequency (enrichment), while negative residuals indicate a lower-than-expected frequency (depletion). Heatmaps illustrate enrichment patterns, with orange indicating enrichment (higher-than-expected frequencies) and blue indicating depletion (lower-than-expected frequencies). The left panels of the heatmaps summarize comparisons across all HC, while the right panels focus on comparisons stratified by HLA-B27 status.

To quantify the effect of AS on the immunophenotype, we compared AS subjects with HC with and without HLA-B27 (**Figure 2**). AS demonstrated a profound impact on gene expression, with 100 genes significantly altered compared to HLA-B27^neg^ HC and an even more substantial effect relative to HLA-B27^pos^ HC, showing 428 altered genes in TNK subsets including *IL-32*, *CD52*, *IL-7R*, *RPS4Y1*, *AHNAK*, and *NEAT1* (**Figure 2A**; **Supplementary Table S5**). The significant overlap in altered gene expression between comparisons with HLA-B27^neg^ and HLA-B27^pos^ HC emphasized disease-specific effects independent of HLA-B27 status, i.e., disease over genotype.

Enrichment analysis using Chi-squared analysis was performed as a high-level measure to compare cellular composition differences across conditions, independent of the number of cells input from each donor. With this approach, we found AS-associated immune programming across multiple immunological lineages. In the TNK compartment, AS subjects showed enrichment of γδT cells, Tregs, CD4 central and effector memory, ILCs, and naive T cells, with concurrent depletion of cytotoxic T cells, MAIT, and NK cells compared to total HC (**Figure 2B**, left panel). Importantly, when controlling for HLA-B27 as a covariate, AS-specific enrichment of γδT cells, Tregs, and ILCs persisted (**Figure 2B**, right panel), indicating these as disease-specific rather than HLA-B27-dependent changes. The B-cell landscape in AS showed characteristic alterations, with expansion of naive B cells and depletion of memory and intermediate B cells and plasmablasts compared to total HC (**Figure 2D**). This was accompanied by upregulation of genes involved in B-cell development and function (*FCER2*, *IGHD*, *IGHM*) and immune regulation (*HLA-DRB6*, *TCL1A*) (**Figure 2C**). Combined, the expansion of naive T and B cells indicates ongoing immune activation and recruitment of new immune cells into the response, whereas the increase in γδT cells and Tregs suggests balancing proinflammatory and anti-inflammatory responses.

The myeloid compartment displayed AS-specific reorganization, characterized by enrichment of CD14 monocytes and depletion of CD16 monocytes, pDCs, and cDC2 cells compared to all HCs (**Figure 2F**, left panel), suggesting a shift toward a proinflammatory myeloid profile. Notably, while most myeloid populations showed disease-specific distributions, the depletion of cDC2 populations was shared between HLA-B27^pos^ AS and HC when compared to HLA-B27^neg^ HC (**Figure 2F**, right panel), suggesting an HLA-B27-associated trait. The AS-specific myeloid signature included upregulation of genes involved in immune regulation (*CD163*, *FKBP5*, *HLA-DRB6*), innate immunity (*IFITM3*, *LGALS2*), and metabolic processes (*RBP7*).

Pathway analysis of DE genes revealed several coordinated programs across cell types. In TNK cells, alterations in immune modulators (*IL-32*, *CD52*, *IL7R*), cellular stress responses (*CMC1*, *H4C3*), and regulatory factors (*NEAT1*) suggest broad reprogramming of T-cell function. B cells showed modulation of transcriptional regulation (*JUNB*, *ETS1*) and RNA processing (*SF3B1*, *SNHG7*) pathways, while myeloid cells exhibited changes in immune regulation (*FKBP5*, *HLA-DRB6*), cell migration (*CX3CR1*), and innate immune function (*FCGR3A*, *CD14*, *CD163*). These coordinated changes across multiple immune compartments suggest AS drives systematic immune reprogramming distinct from HLA-B27-associated effects.

### AAU subjects exhibit distinctive transcriptional suppression and immune cell redistribution with HLA-B27-dependent and HLA-B27-independent effects

**Figure 3:**
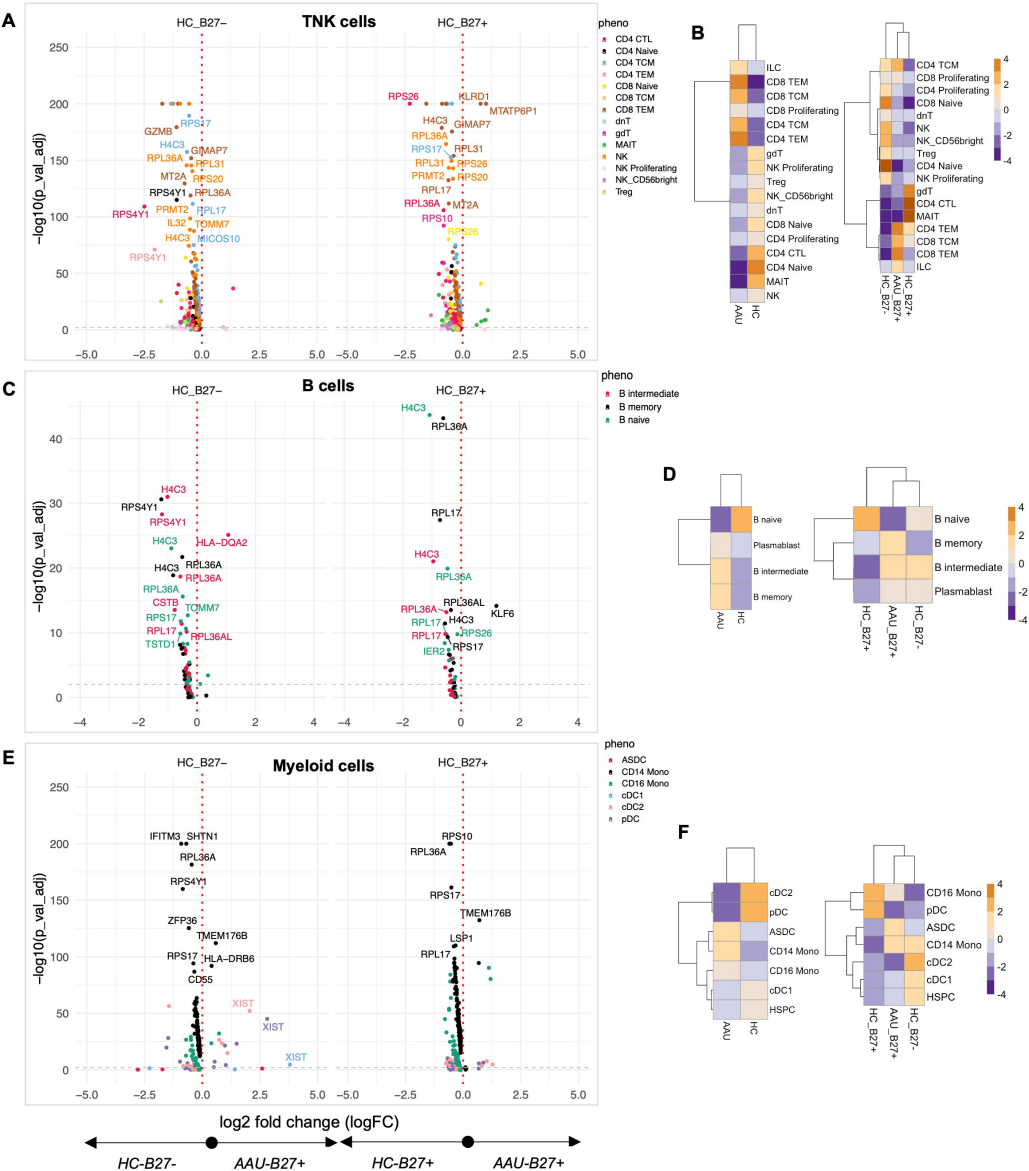
Quantifying the immune landscape in AAU. Subjects with acute anterior uveitis (AAU) were compared to healthy controls (HC) with and without HLA-B27. Differential expression analysis was performed for each major cell lineage: **(A)** TNK cells, **(C)** B cells, and **(E)** myeloid cells. Volcano plots display genes with significant differential expression (FDR-adjusted *p*-value < 0.05). Comparisons to HLA-B27^neg^ HC are shown on the left, while comparisons to HLA-B27-positive HC are shown on the right. Genes with the largest log2 fold changes are uniquely labeled in the plots for each subphenotype. Cellular enrichment for subpopulations within **(B)** TNK cells, **(D)** B cells, and **(F)** myeloid cells was assessed using Chi-squared residuals, calculated from observed vs. expected frequencies. The values displayed in the heatmaps reflect Chi-squared residuals of observed vs. expected frequencies for each cell subset under the indicated conditions. Positive residuals indicate a higher-than-expected frequency (enrichment), while negative residuals indicate a lower-than-expected frequency (depletion). Heatmaps illustrate enrichment patterns, with orange indicating enrichment (higher-than-expected frequencies) and blue indicating depletion (lower-than-expected frequencies). The left panels of the heatmaps summarize comparisons across all HC, while the right panels focus on comparisons stratified by HLA-B27 status.

Since AAU shares a strong association with HLA-B27, we compared AAU subjects relative to HC (with and without HLA-B27). A striking feature across multiple immune compartments was the widespread downregulation of ribosomal proteins. TNK cells from AAU subjects showed coordinated suppression of multiple ribosomal components (*RPS26*, *RPS17*, *RPS4Y1*, *RPS20*, *RPS10*, *RPL27*, *RPL17*, *RPL36A*) compared to all HC (**Figure 3A**; **Supplementary Table S5**), suggesting fundamental alterations in protein synthesis machinery, reflecting a cellular response to stress or an attempt to modulate immune activation through translational control.

The TNK compartment revealed distinct cellular redistribution patterns in AAU. Subjects showed enrichment of CD4 and CD8 CEM and TEM cells, with concurrent depletion of CD4 naive, CD4 cytotoxic, and MAIT cells compared to total HC (**Figure 3B**, left panel). When stratified by HLA-B27 status, AAU subjects maintained a dominant signature enriched with T effector memory and central memory cells (**Figure 3B**, right panel). Notably, AAU subjects showed decreased numbers of γδT, CD4 cytotoxic T, and MAIT cells compared to HC-B27^pos^ HC, while sharing with HC-B27^pos^ HC the depletion of naive and proliferating NK and T cells relative to HC-B27^neg^ HC (**Figure 3B**). The expansion of memory T- and B-cell subsets suggests a heightened adaptive immune response, potentially due to ongoing antigenic stimulation in AAU.

The B-cell compartment exhibited a similar pattern of ribosomal protein suppression, along with decreased expression of *CSTB*, *IFITM3*, *CHST12*, *MS4A1*, and *TOMM2* (**Figure 3C**). However, AAU subjects showed a distinctive expansion of plasmablast, B_inter_, and B_mem_ cells compared to all HCs, an effect primarily driven by comparison with HLA-B27^pos^ HC (**Figure 3D**). This suggests active B-cell differentiation despite suppressed protein synthesis machinery, and the increased plasmablasts further indicate active antibody production, which may play a role in disease pathology. Given that AAU associated with AS is episodic with complete resolution between episodes, the data align with the hypothesis that an antigen-driven event triggers AAU. A similar conclusion was reported by Yang et al. (46).

Myeloid populations reinforced the global pattern of ribosomal protein suppression while showing unique transcriptional features, including increased expression of *TMEM176B* and *HLA-DRB6* compared to all HC (**Figure 3E**). Cellular analysis revealed AAU-specific enrichment of ASDCs, CD14, and CD16 monocytic cells, with concurrent decreases in dendritic cells compared to all HCs (**Figure 3F**, left panel). Interestingly, CD16 monocytic cell patterns showed HLA-B27-dependent effects, with decreased numbers driven by comparison to HLA-B27^neg^ HC, while HLA-B27^pos^ HC showed increased CD16 monocytic cells compared to both AAU subjects and HLA-B27^neg^ HC (**Figure 3F**, right panel). This enrichment of ASDCs and monocytes points toward altered antigen presentation and innate immune activation.

Pathway analysis of the top contributing transcriptional markers revealed coordinated programs across immune compartments. In TNK cells, modulation was observed in cytotoxicity (*KLDR1*, *GNLY*), inflammatory mediators (*IL32*), and cellular stress responses (*H4C3*, *SNRPG*, *MICOS10*). B cells exhibited changes related to protein processing (*CSTB*), cell surface signaling (*MS4A1*), and ribosomal function (*RPL17*, *RPL36A*), while myeloid cells showed alterations in innate immune function (*FCGR3A*, *CD14*), cellular stress responses (*IFITM3*), and protein synthesis (*RPS17*, *RPL36A*). This coordinated suppression of protein synthesis machinery across multiple immune cell types and cell-type-specific alterations in immune programming suggests AAU drives systematic immune reprogramming distinct from healthy controls and HLA-B27-associated effects.

### AS+AAU subjects reveal disease-specific immune signatures with predominant AS-like features

To determine the effect of concomitant AS and AAU on the immunophenotype, we compared subjects with both conditions (AS+AAU) to HC with and without HLA-B27 (**Supplementary Figure S6**). Initial analysis revealed significant alterations in gene expression patterns that overlapped with both AS and AAU signatures across TNK, B, and myeloid cell subsets (**Supplementary Figures S6A, C, E**). Notably, subcellular typing demonstrated that TNK and B-cell profiles in AS+AAU subjects clustered with AS rather than AAU (**Supplementary Figures S6B, D**), while myeloid populations showed clustering among all three disease states (**Supplementary Figure S6F**).

**Figure 4:**
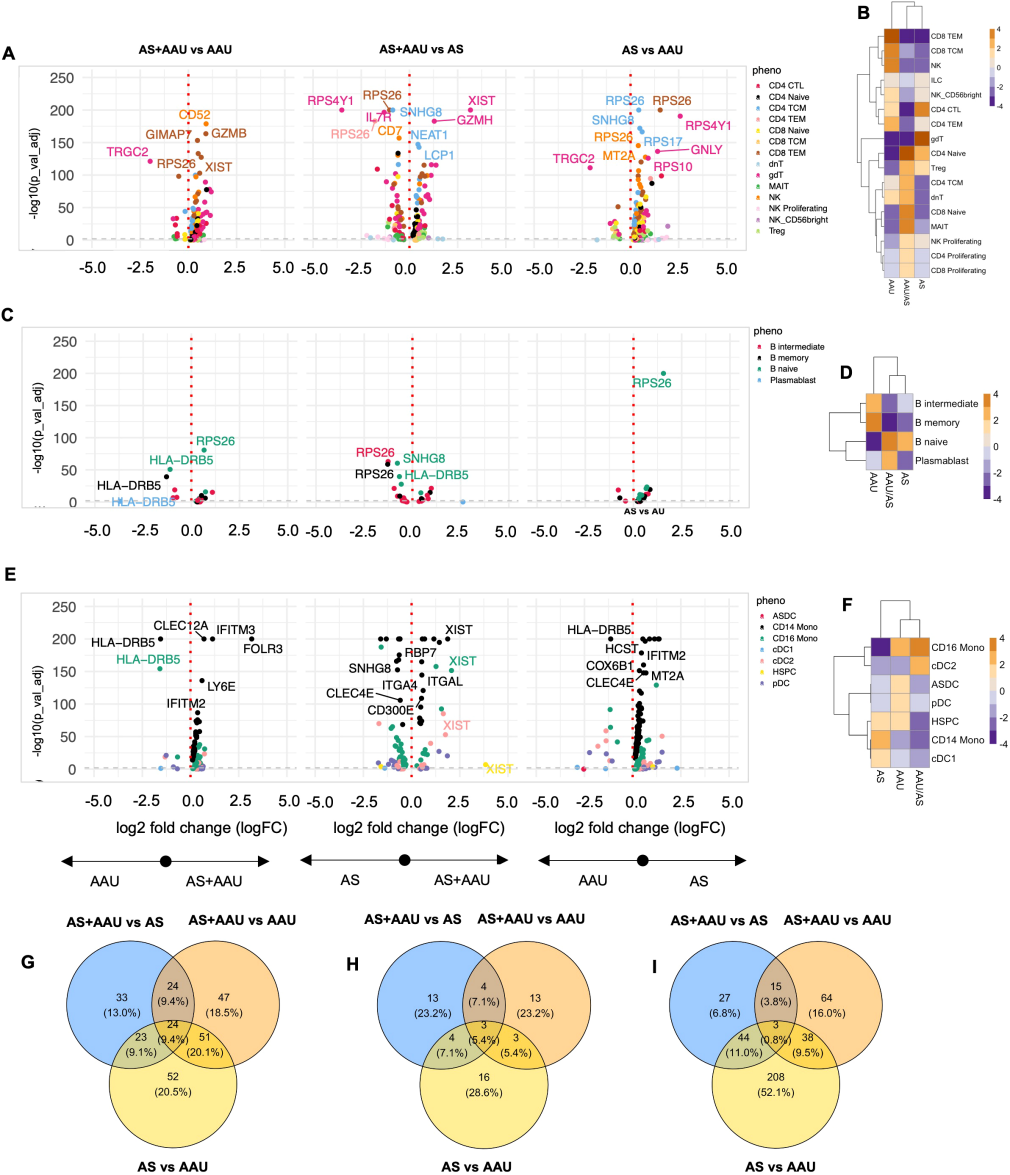
Comparison of AS, AAU, and AAU/AS donors. To complement the previous differential expression analysis at the subphenotype level, we compared three groups of donors: ankylosing spondylitis (AS), acute anterior uveitis (AAU), and those with both conditions (AS+AAU). Differential expression analysis was conducted for each major cell lineage: **(A)** TNK cells, **(C)** B cells, and **(E)** myeloid cells. Volcano plots illustrate genes with significant differential expression (FDR-adjusted *p*-value < 0.05) for the following comparisons: AS+AAU vs. AAU (left), AS+AAU vs. AS (middle), and AS vs. AAU (right). Genes with the largest log2 fold changes are uniquely labeled in each plot for clarity. Cellular enrichment of subpopulations within **(B)** TNK cells, **(D)** B cells, and **(F)** myeloid cells were assessed using Chi-squared residuals, calculated from observed vs. expected frequencies. The values displayed in the heatmaps reflect Chi-squared residuals of observed vs. expected frequencies for each cell subset under the indicated conditions. Positive residuals indicate a higher-than-expected frequency (enrichment), while negative residuals indicate a lower-than-expected frequency (depletion). Heatmaps illustrate these enrichment patterns, with orange indicating enrichment (higher-than-expected frequencies) and blue indicating depletion (lower-than-expected frequencies) across the AS, AAU, and AS+AAU groups. Venn diagrams **(G–I)** summarize the overlap and differences in significantly altered genes between these disease states for **(G)** TNK cells, **(H)** B cells, and **(I)** myeloid cells, highlighting the shared and distinct transcriptional signatures among the conditions.

A detailed comparative analysis between AS, AAU, and AS+AAU subjects (**Figure 4**; **Supplementary Table S5**) revealed distinct disease-specific patterns. In the TNK compartment, AS+AAU subjects showed strong alignment with AS profiles, sharing enrichment of CD4-naive cells, Tregs, and proliferating NK cells, while both groups showed decreased CD4 TEM, TCM, and NK cells compared to AAU subjects (**Figure 4B**). This AS-like signature was further supported by differential expression analysis of AS+AAU versus AAU TNK cells, showing enrichment of cytotoxicity-associated genes (*GZMH*), regulatory factors (*NEAT1*), and cell adhesion molecules (*LCP1*, *ITGB1*, *LGALS1*).

B-cell populations similarly demonstrated AS-dominant features in AS+AAU subjects, with shared enrichment of naive B cells and depletion of intermediate and memory B cells compared to AAU subjects (**Figure 4D**). This pattern was accompanied by increased expression of antigen presentation machinery (*HLA-DQA2*) and transcriptional regulation (*MED13L*). However, the myeloid compartment revealed a contrasting pattern, with AS+AAU subjects clustering with AAU rather than AS profiles. Both AAU and AS+AAU showed increased CD16 monocytes with concurrent depletion of CD14 monocytes and CDC1 cells compared to AS subjects (**Figure 4F**).

Pathway analysis via gene expression overlap revealed distinct patterns across cellular compartments. In TNK cells, substantial condition-specific programming was observed, with each disease state maintaining unique gene signatures and only 10% overlap across all three conditions (**Figure 4G**). B cells displayed even greater disease specificity, with minimal gene expression overlap (3%) between conditions (**Figure 4H**). In contrast, myeloid populations showed a striking dichotomy between AS and AAU signatures, which was partially resolved in the AS+AAU phenotype (**Figure 4I**), indicating distinct myeloid-driven pathogenic mechanisms in AS versus AAU.

These findings reveal that concomitant AS+AAU presents a complex immune signature, characterized by AS-dominant features in adaptive immune compartments (TNK and B cells) while maintaining AAU-like innate immune programming in myeloid populations. This suggests that although adaptive immune responses in AS+AAU primarily follow AS-specific patterns, innate immune mechanisms may be distinctly regulated in the presence of AAU, highlighting the complex interplay between these two HLA-B27-associated conditions. In other words, the divergence between adaptive and innate immune profiles in AS+AAU subjects suggests that the coexistence of AS and AAU may involve additive or synergistic immune mechanisms. Mechanistically, AS-like adaptive immune signature may drive chronic inflammation, while the AAU-like innate immune changes could contribute to acute inflammatory episodes characteristic of uveitis. Understanding the dual immune signatures may inform personalized therapeutic strategies targeting both adaptive and innate immune pathways.

### Uncovering pathological signatures using unsupervised machine learning

To identify pathological signatures in our data, we applied the SDA algorithm, a soft-clustering method that captures transcriptional signatures as components, providing weights for genes (loadings) and rankings for cells (scores); see Material and methods for further details. This section highlights several key findings from the SDA analysis. However, the complete analysis, which includes findings that corroborate known biology and disease pathology described above using our standard analytical approach, is accessible through our interactive website and R package: B27 Ankylosing Spondylitis and Acute Anterior Uveitis Atlas (BASSAA), https://eisascience.shinyapps.io/BASSAA/”.

### AS and AAU share an imbalance in myeloid regulation in comparison with HC

**Figure 5:**
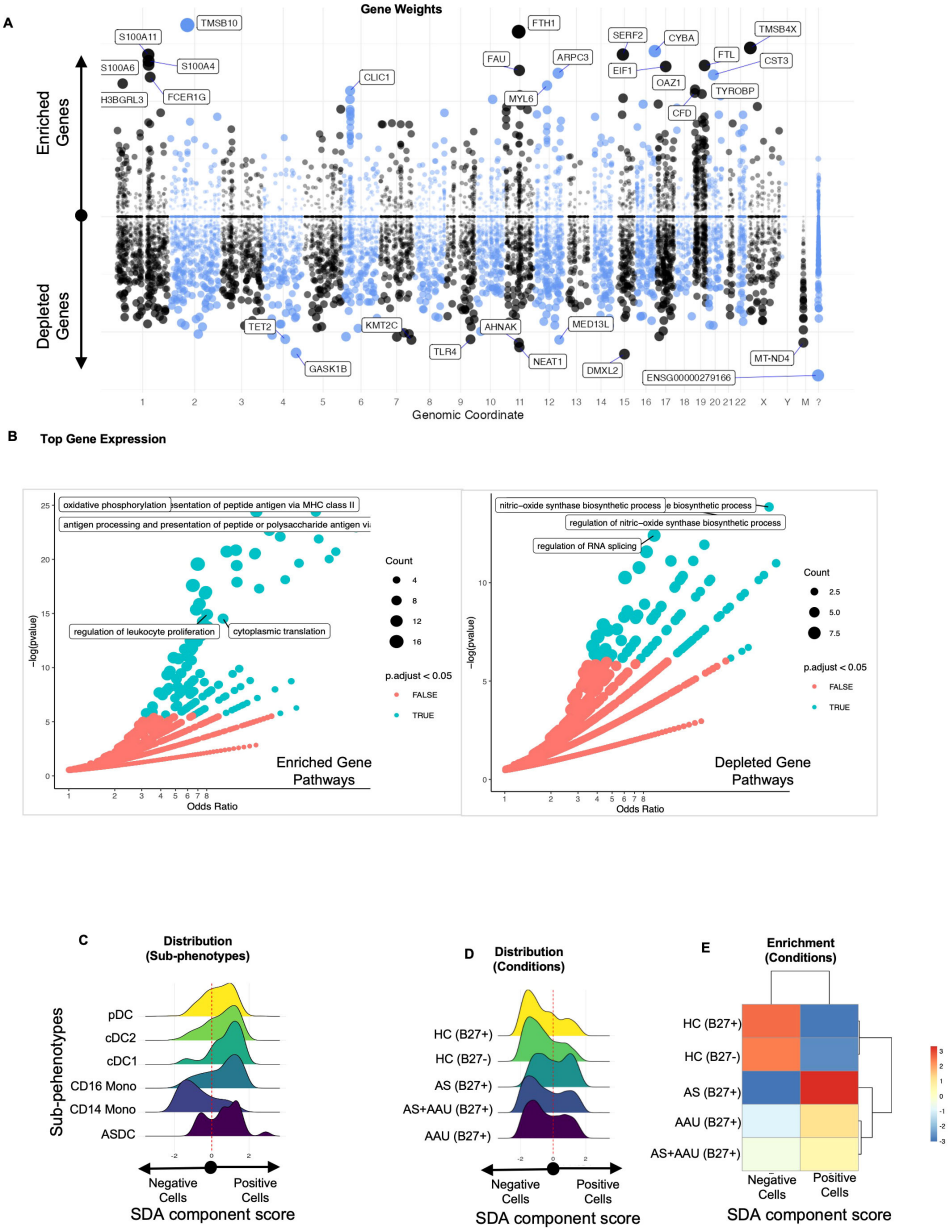
The SDA component (SDA48) associated with myeloid regulation imbalance (MRI) implicates myeloid cell activation and metabolic homeostasis as underlying factors in both AS and AAU. We performed an SDA analysis and identified a myeloid regulation imbalance (MRI) component—a unique gene signature that distinguishes inflammatory immune activation (positive) from metabolic and signal regulation (negative). The gene loadings for this component are shown in **(A)**, sorted by their mapping locations on the human chromosome. The top-weighted genes in positive and negative directions are shown. The top-loaded genes were used for GO enrichment (see Material and methods), and the results are visualized in **(B)**, highlighting significant potential matches. In **(C)**, the distribution of these cells within this component is shown, stratified by immune subphenotypes, while **(D)** displays the distribution across disease conditions. To quantify the differences in these distributions, we assessed the enrichment of cells that scored either positively or negatively in **(E)**.

One of the SDA components (SDA component 48) identifies a myeloid activation and metabolic homeostasis signature leading to myeloid regulation imbalance (MRI). This signature highlights an immune imbalance with increased activation of dendritic cells and CD16^+^ monocytes, along with a decrease in genes required to maintain the immune cell homeostasis (normal cellular function of immune cells to maintain a balance between identification and active elimination of foreign pathogens while preventing response to self-antigens) in NKT cells. This pattern of gene expression defines a loss in function in managing appropriate cellular functions such as mounting an appropriate immune response to stimuli while maintaining the identity of self (47, 48). The top positive driving (enriched) genes (*TMSB10*, *FTH1*, *TMSB4X*, *CYBA*, *SERF2*, *S100A11*, *S100A4*, *S100A6*, *FTL*, *EIF1*, *FAU*, *ARPC3*, *CST3*, *FCER1G*, *SH3BGRL3*, *MYL6*, *OAZ1*, *CLIC1*, *CFD*, and *TYROBP*) (**Figure 5A**) are associated with an active immune/inflammatory response, cytoskeletal organization, and stress management. High scores indicate cells actively presenting antigens, responding to immune signals, and maintaining structural integrity (**Figure 5B**). This is typical of dendritic cells (DCs) and CD16^+^ monocytes, which are crucial in initiating and sustaining immune responses. The enrichment of these cells in AS and AAU supports the known pathology of ongoing inflammation and immune activation.

On the other hand, the top negative driving (depleted) genes (*DMXL2*, *GASK1B*, *NEAT1*, *AHNAK*, *MT-ND4*, *KMT2C*, *MED13L*, *TLR4*, *TET2*, *CD36*, *TMEM170B*, *GAS7*, *RBM47*, *SPOPL*, *KDM7A*, *TFEC*, *PLXDC2*, *TAOK1*, *MT-ND2*, *OGFRL1*, *ZSWIM6*, *BAZ2B*, *MEGF9*, and *VCAN*) reflect metabolic processes, mitochondrial function, and transcriptional regulation (**Figure 5A**). These upregulated/enriched (**Figure 5B**, left panel) genes belong to inflammatory pathways like antigen presentation to MHC, antigen processing and presentation of peptides and polysaccharide antigens, oxidative phosphorylation, regulation of leukocyte proliferation, and cytoplasmic translation, while the downregulated/depleted genes (**Figure 5B**, right panel) belong to metabolic and regulatory functions such as regulation of RNA splicing and regulation of nitric oxide biosynthetic processes(**Figure 5B**). These results are observed in dendritic cells and monocytic cells (**Figure 5C**), including pDCs, which can produce high levels of interferons, and ASDCs, which have a high potential for T-cell activation (49). High scores correspond to cells maintaining homeostasis, managing oxidative phosphorylation, and regulating gene expression. This function and gene signature are typical of CD14^+^ monocytes, which are more involved in phagocytosis and metabolic regulation, with less immediate immune activation. Cells with negative scores are enriched in healthy controls, indicating that AS and AAU present a myeloid landscape shifted from the normal metabolic and regulatory functions of CD14^+^ monocytes toward an inflammatory state driven by CD16^+^ monocytes and DCs (**Figures 5D, E**). All genes contributing to the SDA component MRI are detailed in **Supplementary Table S6**.

### AS and AAU exhibit a differential pDC-monocyte signature, characterized by monocyte enrichment and concurrent depletion of DCs in their PBMCs

Building upon our previous findings, we found another SDA component (SDA component 65) associated with the activation of pDCs, which serves as a myeloid cell signature distinguishing DCs from monocytes. In this component, pDCs exhibited the highest positive scores, followed by other DC subsets in the positive direction, while CD14^+^ and CD16^+^ monocytes scored negatively. We found that regardless of the HLA-B27 status, the HCs were enriched with DCs, whereas AS and AAU were enriched with monocytes (**Supplementary Figure S6F**). The top positive weighted (enriched) genes (*CLEC4C*, *LILRA4*, *SERPINF1*, *SCT*, *PTPRS*, *LRRC26*, *PTCRA*, *MAP1A*, *PLD4*, *DERL3*, *DNASE1L3*, *ITM2C*, *TPM2*, and *SMPD3*) strongly indicate the presence of DCs, especially pDCs. In contrast, the negative weighted (depleted) genes (*TMSB4X*, *TMSB10*, *TXNIP*, *PTPRC*, *KLF2*, *CD52*, *COTL1*, *FTL*, *HLA-A*, *S100A4*, *CD48*, *YBX3*, *FCN1*, *CFD*, *SERPINA1*, *FGL2*, *GIMAP4*, *ACTB*, *HLA-E*, *LGALS2*, *LGALS3*, *CPVL*, *AIF1*, *MNDA*, *HLA-B*, *NEAT1*, *ZFP36L1*, *CD14*, *MT-CO3*, and *LYZ*) indicate a generalized monocyte signature (**Supplementary Figure S8A**). All genes contributing to this SDA component (pDC-monocyte differential signature [PMDS]) are detailed in **Supplementary Table S6**. Their enrichment in AS and AAU suggests an active inflammatory response in these conditions.

Combined, this component highlights the restriction of pDCs and ASDCs in AS and AAU, which may contribute to a failure in regulating the immune response, hindering T-cell regulation, and leading to chronic inflammation and autoimmunity (**Supplementary Figure S8B**). This was highlighted by the upregulation of pathways for immune response-activating signaling, regulation of response to biotic stimuli, and regulation of immune effector processes (**Supplementary Figure S8B**, left panel) with the downregulation of dendritic cell development (**Supplementary Figure S8B**). The increased monocyte-associated genes and the concomitant decrease of DC-associated genes in AS and AAU subjects suggest an active inflammatory response characterized by monocyte enrichment and DC depletion. Further analysis demonstrated that the positive SDA scores were primarily driven by DC subtypes—pDCs, conventional dendritic cells type 1 (cDC1), cDC2, and ASDCs—rather than by the disease conditions themselves (**Supplementary Figures S8C, D**). The deficiency of pDCs and other DCs in AS and AAU suggests a disruption in the balance between proinflammatory and regulatory mechanisms within the immune system, favoring the enrichment of proinflammatory myeloid cells such as monocytes and macrophages (**Supplementary Figure S8E**). This imbalance may lead to an unchecked inflammatory response, contributing to the pathogenesis of AS and AAU.

### Distinct B-cell defect signature in AS subjects

**Figure 6:**
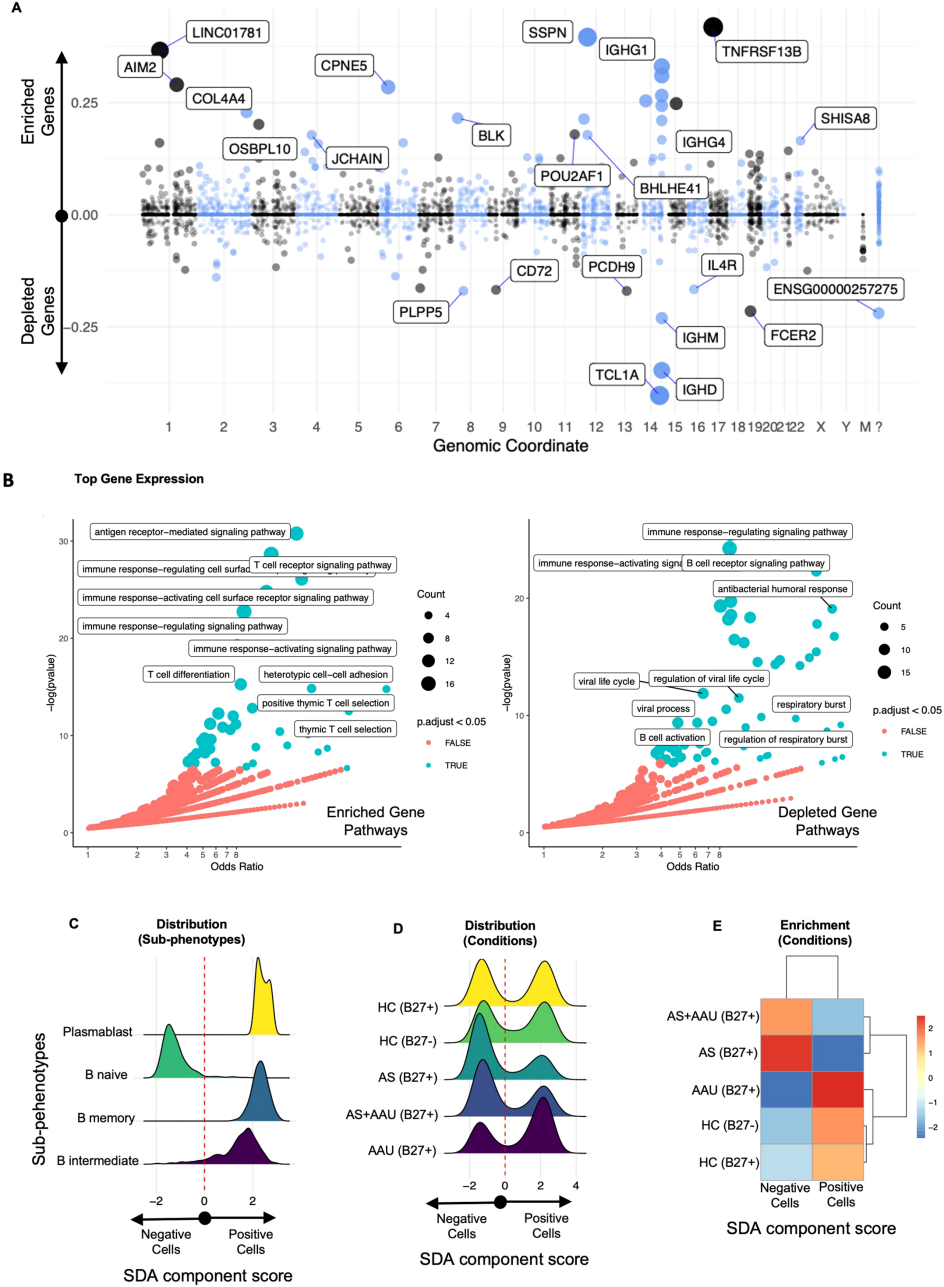
The SDA component (SDA 76) B-cell defect signature (BCDS) differentiates disease pathogenesis between AS and AAU subjects. This component features a unique gene signature that distinguishes naive B cells (negative) from memory and intermediate B cells, as well as plasmablasts (positive). Moreover, this signature identifies AS—but not AAU—as enriched with naive B cells, suggesting potential dysregulation in B-cell maturation and class switching. The gene loadings for this component are shown in **(A)**, sorted by their mapping locations on the human chromosome. The top-weighted genes in positive and negative directions are shown. The top-loaded genes were used for GO enrichment (see Material and methods), and the results are visualized in **(B)**, highlighting significant potential matches. The distribution of these cells, as scored by this component, is displayed in **(C)** for immune subphenotypes and in **(D)** for disease conditions. To quantify the differences in these distributions, we performed an enrichment assessment of cells that scored either positively or negatively in **(E)**.

While both AS and AAU were associated with higher immune/inflammatory pathways (**Figure 5**), we further explored whether AS and AAU were immunophenotypically distinct. Indeed, we found multiple SDAs that differentiated between AS and AAU (**Figure 6**; **Supplementary Figure S9**).

One notable SDA component (SDA component 76), referred to as B-cell defect signature (BCDS), distinguishes between mature, class-switched B cells (memory B cells and plasma cells) on the positive side and naive or early-stage B cells on the negative side (**Figure 6A**). Higher positive values (enriched genes) indicate a higher presence of mature B cells with roles in adaptive immunity through pathways like antigen receptor-mediated signaling, T-cell receptor signaling, etc., characterized by the expression of specific genes (such as *TNFRSF13B*, *SSPN*, *LINC01781*, *IGHG1*, *IGHA2*, *AIM2*, *CPNE5*, *IGHG3*, *COCH*, *TEX9*, *IGHA1*, *COL4A4*, *BLK*, *CLECL1P*, and *IGHG2*). Conversely, on the negative side, the depleted genes represent naive and early-stage B cells (characterized by genes such as *TCL1A*, *IGHD*, *IGHM*, *FCER2*, *PCDH9*, *PLPP5*, *CD72*, *IL-4R*, and *ACTB*), leading to B-cell receptor pathways, antibacterial humoral response, B-cell activation, etc. (**Figure 6B**, detailed gene list in **Supplementary Table S6**). The upregulated/enriched genes (**Figure 6B**, left panel) are associated with immunological pathways such as antigen receptor-mediated signaling, T-cell receptor signaling, immune response activating cell surface receptor signaling, T-cell differentiation, and positive thymic T-cell selection, among others. In contrast, the downregulated/depleted genes (**Figure 6B**, right panel) are involved in pathways related to the dampening of B-cell and immune functions, including B-cell receptor signaling, antibacterial humoral response, B-cell activation, immune response activating/regulating response, and viral processes (**Figure 6B**).

The full score clearly distinguishes naive B cells as negatively scored and other B cells and plasmablasts as positive (**Figure 6C**). This score identifies a bimodal distribution relative to all conditions (**Figure 6D**). Thresholding this score identifies the negatively scored cells that are enriched in AS, whereas the positive cells are enriched in AAU and HC, such that AS and AAU show the largest enrichment differential. AS+AAU aligned with AS (**Figures 6D, E**), further confirming our data for a dominant AS signature in these subjects (**Figure 4**). This signature suggests a potential mechanism that involves *IL-4R*, *TCL1A*, and *CD72* as mediators in signaling pathways for B-cell development and activation.

### Enrichment of TNK cytotoxic module in AAU and HLA-B27^pos^ HC

In our ongoing exploration of immunophenotypic differences, we identified an SDA component (SDA component 27) associated with cytotoxic TNK cells (TCM). The top positive/enriched genes (such as *CCL5*, *IL-32*, *GZMH*, *B2M*, *ITGB1*, *S100A4*, *SH3BGRL3*, and *KLRG1*) in this component indicate the activity of cytotoxic T and NK cells (**Supplementary Figure S9**). These cells play a crucial role in the direct elimination of infected or abnormal cells, antigen presentation, and recruiting other immune cells to sites of inflammation. Conversely, the top negative/depleted genes (such as CCR7, LEF1, SELL, IL-6ST, TXK, TCF7, CD7) suggest the presence of naive and central memory T cells (**Supplementary Figure S9A**, detailed gene list in **Supplementary Table S6**). GO enrichment analysis of these genes (**Supplementary Figure S9B**) revealed that the positively enriched genes are involved in pathways associated with cytotoxic T cells, such as cell killing, leukocyte-mediated immunity, mononuclear cell differentiation, T-cell receptor signaling, and T-cell-mediated cytotoxicity (**Supplementary Figure S9B**, left panel). In contrast, the negative/depleted genes correspond to metabolic pathways like cytoplasmic protein translation (**Supplementary Figure 9B**, right panel).

Although gene expression does not distinguish major differences between the conditions, the score distribution across the TNK cells, as part of a finely tuned multigene signature, differentiates naive and central memory cells (negatively scored) from effector and cytotoxic TNK cells (positively scored). This signature encapsulates multiple cell types (**Supplementary Figure S9C**), but a differential score distribution is observable across the conditions (**Supplementary Figure S9D**). Counting the cells in each condition that score positive or negative, we observe that the activated cytotoxic signature is enriched in HLA-B27^pos^ and AAU and depleted in AS and HLA-B27^neg^ HCs. Conversely, the negative naïve and central memory cells are enriched in AS and HLA-B27^neg^ HCs and depleted in HLA-B27^pos^ and AAU (**Supplementary Figure S9E**).

These findings suggest that individuals expressing the HLA-B27 molecule exhibit higher levels of activated cytotoxic TNK cells, which could play a role in immune surveillance or predispose them to inflammatory responses observed in AAU. Interestingly, the distribution of naive versus activated cytotoxic TNK cells in AS subjects resembles that of HLA-B27^neg^ HC, indicating a relative depletion of cytotoxic TNK cells in AS, despite the presence of HLA-B27. In contrast, AAU subjects resemble HLA-B27^pos^ HC, with an enrichment of activated cytotoxic TNK cells. Globally, this differential distribution identified by this component’s score reflects the distinct immunopathological mechanisms between AS and AAU. The enrichment of cytotoxic TNK cells in AAU and HLA-B27^pos^ HC is likely associated with heightened cytotoxic immune responses, potentially contributing to ocular inflammation characteristic of AAU. The relative depletion of these cells in AS suggests alternative pathogenic pathways, possibly involving other immune cell types or regulatory mechanisms.

### SDA components identified were validated using the published single-cell CITE-Seq dataset

**Figure 7:**
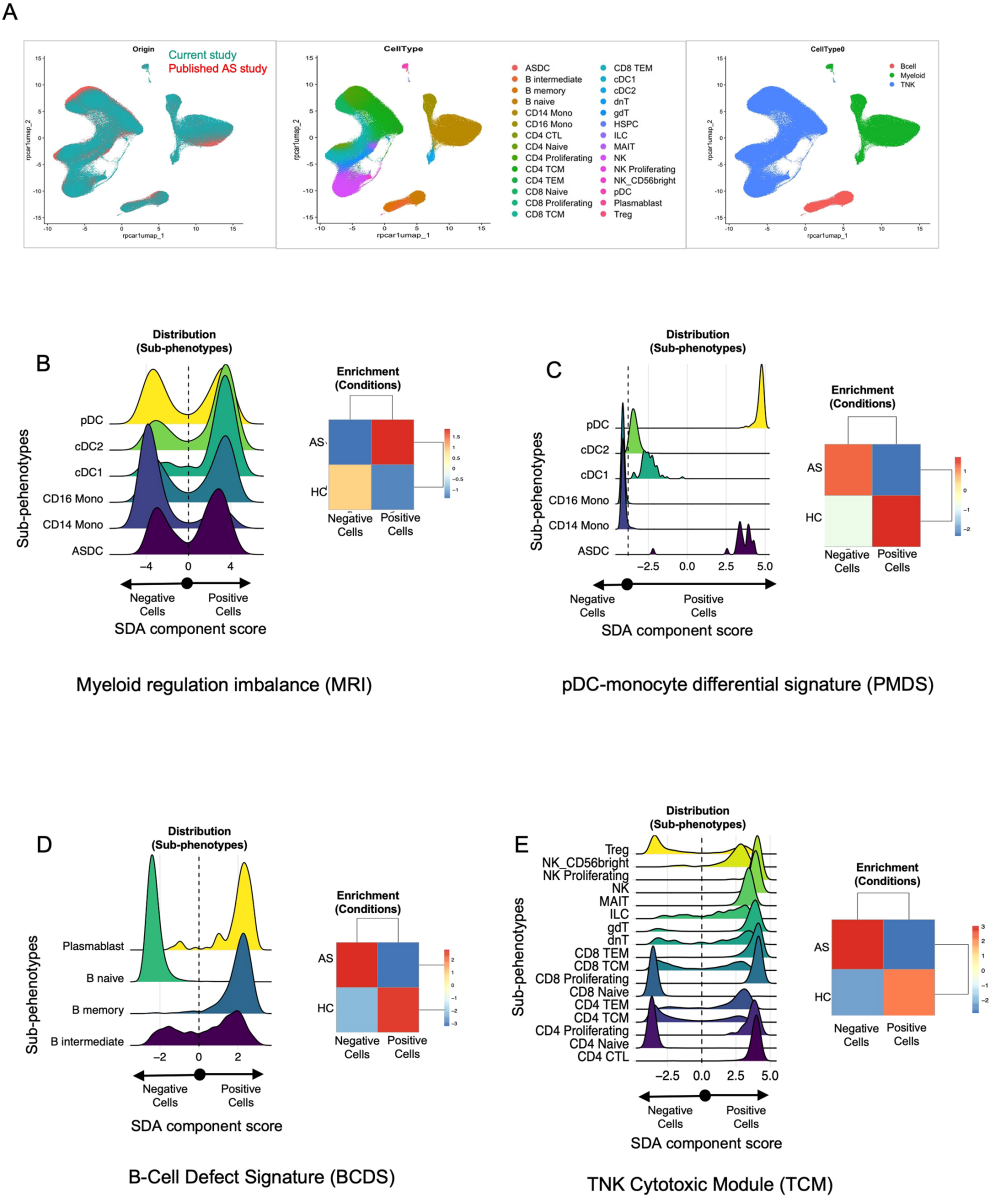
Validation of SDA components identified in this study using a published dataset. **(A)** Cross-study UMAP overlap. Single-cell data from our CITE-Seq study (teal, left panel) and the published AS dataset (orange, left panel) were integrated and visualized using UMAP. The middle panel shows the cellular subclusters (*N* = 28), while the right panel presents cellular clustering by broad cell type (e.g., B cells, myeloid cells, TNK cells) across both datasets. These figures highlight the cellular overlap between our dataset and the published dataset, enabling projection of SDA components identified in our dataset onto the published dataset. **(B–E)** Distribution plots and enrichment heatmaps. Each panel represents one of the four sparse decomposition analysis (SDA) components identified as relevant in our study: **(B)** myeloid regulation imbalance (MRI), **(C)** pDC–monocyte differential signature (PMDS), **(D)** B-cell defect signature (BCDS), and **(E)** TNK cytotoxic module (TCM). In each figure **(B–E)**, the left panel displays cell distribution plots, where the *x*-axis represents the SDA component “score” for each cell, derived from the underlying gene set. Cells are categorized as “negative” vs. “positive” based on whether their component score falls below or above zero. Cells with higher scores (to the right) exhibit stronger expression of the genes driving the SDA component, whereas those with lower scores (toward the left) express those genes at reduced levels. The colored curves represent the distribution of component scores within different subphenotypes (e.g., cDC1, pDC, CD14 Mono). Peaks shifted to the right indicate greater expression of the component’s gene set within that subphenotype. Likewise, in each figure **(B–E)**, the right panel displays the enrichment of these cells in AS vs. HC from the published datasets. These heatmaps illustrate whether AS or HC samples are overrepresented (enriched) or underrepresented (depleted) among cells with positive vs. negative scores. The numeric scale (e.g., − 3 to + 3) represents standardized residuals (e.g., *χ* ^2^ residuals or *Z*-scores). Positive values (red) indicate enrichment, while negative values (blue) indicate depletion of that subset or condition beyond what is expected by chance. These values were calculated using a Chi-squared test (or a similar enrichment test) by comparing observed vs. expected cell frequencies in each group. Detailed methods, including significance thresholds and any corrections for multiple comparisons, are provided in the Material and methods section. Taken together, panels **(B–E)** confirm that the SDA components identified in our main dataset are recapitulated in an independent, published AS cohort, highlighting specific immune subsets (e.g., myeloid cells, pDCs, B cells, T/NK cells) that exhibit disease-associated signature scores in AS vs. HC.

To ensure the robustness and generalizability of findings in our SDA analysis, we validated them by using a previously published dataset (27) on AS subjects and HC. First, by similar reprocessing of the previously published data followed by integration with our data, we were able to confirm that both PBMC single-cell CITE-Seq datasets were overlapping in terms of their cell type distributions, including our classification of TNK, B, and myeloid cell groups (**Figure 7A**). Next, we projected all the components from our trained SDA model onto the published data, which computed cell scores in this new dataset for each component. Indeed, we found these components, including those highlighted herein, to have similar cell distribution patterns across disease categories and associated cell types, including the myeloid regulation imbalance (MRI; **Figure 7B**, also see **Figure 5**), (**Figure 7C** (PMDS), also see **Supplementary Figure S8**), the B-cell defect signature (**Figure 7D** (BCDS), also see **Figure 6**), and the TNK cytotoxic module (**Figure 7E** (TCM), also see **Supplementary Figure S9**). This validation study strengthens our conclusions, eliminates a potential small cohort size bias, and provides a broader understanding of the distinct and shared pathophysiology underlying AS and AAU.

## Discussion

This study entails immunophenotype characterization of PBMCs from HLA-B27^pos^ AS, AAU, and AS+AAU subjects in comparison with HC (with and without HLA-B27). To our knowledge, this is the first study to compare and determine immunophenotypes for AS and AAU, as well as identify the effect of HLA-B27, a strong genetic risk factor for AS and AAU (9, 10), and its effect on HC. Although HLA-B27 in HC is associated with changes in the gut microbiota, its effect on the host immune response remains unclear (50). In addition to host genetics, we identified disease-associated immunophenotypes in both AS and AAU. Notably, AS+AAU subjects showed a closer phenotypic resemblance to AS, suggesting a stronger impact of AS on the immunophenotype. This finding parallels a study comparing gut microbial dysbiosis, where the gut microbiota of AAU patients with SpA was found to be more similar to that of SpA-only patients than to HCs (51). Furthermore, SDA analysis revealed that both share certain inflammatory pathways, while also exhibiting distinct immune profiles and pathological signatures, shedding light on the signatures that may drive the progression of each disease.

Our analysis revealed that HLA-B27 expression in HC significantly alters the immunophenotype, even in the absence of disease. HLA-B27^pos^ HC showed enrichment of cytotoxic and effector T cells, including MAIT cells and γδT cells, along with increased CD16^+^ monocytes and pDCs. These changes suggest a baseline shift toward heightened innate immune surveillance and cytotoxic potential. While HLA-B27 has previously been linked to altered gut microbiota composition in HC (26), our findings extend this knowledge by demonstrating its impact on immune cell populations. This altered immunological baseline may predispose HLA-B27^pos^ individuals to inflammatory conditions such as AS and AAU.

Specifically, we observed alterations in various cellular subsets, including TNK, B, and myeloid cells in AS subjects. Similar to previous studies showing a decrease in NK cells (28), we observed a decrease in NK, NK proliferating, and NK CD56^bright^ cells compared with all HC. However, when considering HLA-B27, these differences were only evident when compared to HLA-B27^neg^ HC, suggesting that HLA-B27 affects the immunophenotype. Similarly, a decrease in various NK cell subsets was observed in AAU, further emphasizing the shared immunopathology between AS and AAU. AAU was also associated with the proliferation of CD4 and CD8 subtypes in the PBMCs, which correlates with an increase in CD4 and CD8 populations in the aqueous humor and PBMCs in various types of human uveitis (19) and correlates with a recent study reporting the expansion of CD8 clones restricted to certain autoantigens presented by HLA-B27 (52).

Since the microbiota plays an important role in disease development in both AS (21, 22) and AAU (51), we found that MAIT cells (CD161^hi^, Cd69^hi^, TCR Va 7.2^hi^) were significantly decreased in both AS and AAU subjects in comparison with HC. Similar observations have been reported in AS, IBD, and autoimmune uveitis (53–55).

Both AS and AAU were associated with common activation of immune/inflammatory pathways with a concomitant decrease in cellular metabolic function. In addition, both AS and AAU showed enrichment of cytotoxic and effector T cells, which overlapped with HLA-B27^+^ HC, highlighting the role of HLA-B27 in altered immunophenotypes. Furthermore, SDA component MRI identified a myeloid cell-associated signature with genes indicative of active immune responses, cytoskeletal organization, and stress management, typical of DCs and CD16^+^ monocytes. These cells, essential for initiating and sustaining immune responses, were enriched in AS and AAU, supporting the known pathology of ongoing inflammation and immune activation. Conversely, the negative genes, associated with metabolic and transcriptional regulation, were more typical of CD14^+^ monocytes and were enriched in HC. This shift from CD14^+^ monocytes to inflammatory CD16^+^ monocytes and DCs in AS and AAU highlights a myeloid landscape geared toward inflammation rather than homeostasis. These CD14^low^/CD16^+^ nonclassical monocytes are inflammatory in response to TLR stimulation; however, they express a remarkably high basal level of miR-146a, a microRNA known to negatively regulate the TLR pathway, thereby contributing to inflammation (56). Interestingly, this SDA component revealed TLR4 as the top gene with decreased expression, further supporting this notion. Furthermore, another SDA component acts. pDCs highlighted a myeloid cell signature distinguishing DCs from monocytes. While pDCs and other DC subsets were enriched in HC, CD14, and CD16 monocytes were more prevalent in AS and AAU. CD16 monocytes have an inflammatory role, and their expansion has been associated with AS, AAU, and other inflammatory diseases (57–59). The pDCs play an important role in immune regulation and antiviral defense, and aberrant distribution and function of pDCs in PBMCs and inflamed synovium of AS subjects is association with unfolded protein response due to enhanced expression of pDC trafficking molecules, CCR6 and CCL20 (60). The ASDC has only recently been identified in humans and exhibits a high capability for T-cell activation (49). The depletion of DCs in AS and AAU suggests a failure in properly regulating the immune response, leading to chronic inflammation and autoimmunity. The imbalance favoring proinflammatory monocytes over regulatory DCs could result in inadequate antigen presentation and persistence of autoreactive T cells, contributing to the pathology of AS and AAU. Taken together, HLA-B27^pos^ individuals exhibit enhanced cytotoxic and innate immune responses, including increased activity of NK cells and CTLs, which may drive excessive inflammation in AS and AAU. These immune alterations, coupled with HLA-B27 misfolding and ER stress, promote proinflammatory cytokine production, such as IL-17 and IL-23, heightening susceptibility to inflammatory diseases. Even in healthy HLA-B27^pos^ individuals, subclinical immune changes can predispose them to AS and AAU, with triggering events such as infections potentially initiating disease. These overlapping immune responses in AS and AAU as well as HLA-B27^+^ HC may play a role in the concomitance of AS and AAU, especially in the presence of HLA-B27.

In contrast to overlapping immunophenotypes in AS and AAU subjects, SDA analysis also uncovered distinct pathological signatures in immune cell populations across HLA-B27^pos^ AS and AAU, which imply their distinct underlying pathophysiology. SDA component BCDS identified a B-cell signature, which represented an enrichment of mature, class-switched B cells associated with decreased numbers and magnitude of naive or early-stage B cells. The positive genes, including *TNFRSF13B* and *IGHG1*, indicated B^mature^ cell functions crucial for adaptive immunity. This signature revealed that mature B cells were enriched in HC and AAU, whereas AS subjects exhibited a higher proportion of naive B cells. Increased levels of naive B cells and decreased levels of differentiated B cells (plasmablasts and B memory cells) have been previously reported in AS subjects (61, 62). This impaired maturation may contribute to a skewed immune profile and chronic inflammation in AS (63). While B cells are involved in the pathogenesis of autoimmune diseases through autoantibody production and regulating T-cell responses via antibody-independent functions, their role in AS and AAU pathogenesis has been unclear (64, 65). A recent study has shown aberrant activation of B-cell populations in AS subjects (66). AAU subjects may have a localized immune response without the systemic immune dysregulation seen in AS, which may point to the B-cell difference in AS and AAU pathogenesis despite the common HLA-B27 association.

In addition, the SDA TCM revealed a signature enriched in cytotoxic T and NK cells, characterized by genes such as *CCL5*, *IL-32*, *GZMH*, and *KLRG1*. These cells are involved in direct cytotoxic activities, antigen presentation, and recruitment of other immune cells. IL-32 is an inflammatory cytokine, which can induce the expression of TNF, IL-1, and IL-6 and chemokines (67). Among these cytokines, TNF is associated with increased inflammatory cell infiltration (68), while IL-1 plays a crucial role in TH17 cell differentiation and disease initiation (69). Blocking IL-6 has been shown to mitigate uveitis (70, 71) in experimental autoimmune uveitis models. Additionally, CCL5, a chemokine with increased expression in the uvea, may contribute to AAU by recruiting inflammatory cells to the eye (72, 73). Interestingly, this cytotoxic activation signature was enriched in AAU and HLA-B27^pos^ subjects, while being depleted in AS and HLA-B27^neg^ HC. The negative side of the signature, represented by genes such as *CCR7* and *LEF1*, indicates the presence of naive and central memory T cells. This suggests differential regulation of TNK cells, where AAU mirrors the activated cytotoxic profile of HLA-B27^pos^ HC, whereas AS exhibits a naive profile similar to HLA-B27^neg^ HC. These findings highlight the potential role of HLA-B27 in modulating TNK cell activation and suggest a protective cytotoxic response in HLA-B27^pos^ HC and AAU. However, AS and AAU also exhibit distinct immune phenotypes that drive their respective disease processes.

Taken together, our data suggest that AS and AAU share certain immune and inflammatory pathways, particularly the enrichment of cytotoxic and effector T cells, as well as inflammatory CD16^+^ monocytes, highlighting the role of HLA-B27 in their altered immunophenotypes and the promotion of chronic inflammation. However, distinct immune signatures may play a key role in differentiating AS from AAU. AS is characterized by a skewed B-cell profile, with an increased proportion of naïve B cells and impaired maturation, whereas AAU exhibits a more localized immune response with a mature B-cell signature. Additionally, cytotoxic activation of TNK cells is enriched in AAU and HLA-B27 ^pos^ HC but depleted in AS, suggesting a differential regulation of cytotoxicity. Overall, these findings suggest that while AS and AAU share an HLA-B27-associated inflammatory environment required for chronicity, their distinct immunophenotypic alterations may underlie differences in disease development and progression.

### Limitations of the study

The absence of HLA-B27^neg^ AS and AAU subjects in our study limits the ability to fully assess the role of HLA-B27 in disease pathogenesis. Future studies including HLA-B27^neg^ AS and AAU subjects would allow comparative analyses to distinguish HLA-B27-associated disease features from those mediated by other genetic or environmental factors. This could provide a deeper understanding of whether the enhanced cytotoxic and innate immune features observed in our study are exclusive to HLA-B27 or part of broader inflammatory mechanisms. Another limitation of this study is the small sample size (*N* = 5/group). Although we selected well-characterized samples from all patient groups and validated our findings using independent analysis of a previously published dataset, larger sample sizes in future studies will be beneficial. Further research is needed to unravel these complex interactions and account for the heterogeneity across multiple disease groups. Developing gene profiles that reflect the disease stratification could enhance our understanding of cellular phenotypes contributing to AS or AAU. Additionally, our study was limited in assessing gender/sex differences across contrasts; future studies with higher cohorts and dedicated methodologies are necessary to better understand the role of sex in autoimmune diseases such as AS and AAU.

In conclusion, using single-cell CITE sequencing, we identified shared and unique immunophenotypic biomarkers associated with disease (AS and AAU) and host genetics (HLA-B27). These findings may contribute to improved disease stratification and the advancement of precision medicine in AS and AAU.

## Data Availability

The datasets presented in this study can be found in online repositories. The names of the repository/repositories and accession number(s) can be found in the article/**Supplementary Material**.
