# Supplementary material for: Divergent B-cell and cytotoxic TNK cell activation signatures in HLA-B27-associated ankylosing spondylitis and acute anterior uveitis
Source: Front Immunol. 2025 Mar 7;16:1546429. doi: 10.3389/fimmu.2025.1546429 (PMC11926545; doi:10.3389/fimmu.2025.1546429)
Supplement: Supplementary file 1 [file DataSheet1.docx]

**Supplementary Tables**

## Supplementary Table 1. General characteristics of the subjects providing blood samples for single cell CITE-Seq analysis between AS, AS+AAU and AAU subjects versus HC.

**Supplementary Table 2**: HLA-B27 allele subtype status for the study cohort.

## Supplementary Table 3: Number of QC-passing cells in each condition/contrast.

**Supplementary Table 4**: Number of QC-passing genes in each subtype.

**Supplementary Table 5:** HLA-B27 and disease associated DE genes in cellular subtypes.

**Supplementary Table 6**: SDA components along with their top-loaded genes.

Supplementary Tables 1-4 are Word files

Supplementary Tables 5-6 are Excel files

## Supplementary Table 1. General characteristics of the subjects providing blood samples for single cell CITE-Seq analysis on AS, AAU, AS+AAU and HC.

|  | **AS** | **AAU** | **AS+AAU** | **HC**  **(HLA-B27^pos^)** | **HC**  **(HLA-B27^neg^)** |
| --- | --- | --- | --- | --- | --- |
| **Age, mean (range)** | 41.8 (19-65) | 52.8 (35-65) | 44.8 (30-54) | 50.2 (25-64) | 40 (25-53) |
| **Female** | 2 (40%) | 3 (60%) | 3 (60%) | 3 (60%) | 2 (40%) |
| **Race** |  |  |  |  |  |
| **White** | 5 | 5 | 4 | 4 | 3 |
| **Asian** | 0 | 0 | 1 | 0 | 0 |
| **Other or >1 race** | 0 | 0 | 0 | 1 | 2 |
| **Hispanic ethnicity** | 0 | 1 | 1 | 0 | 0 |
| **BMI, mean ± SE kg/m^2^** | 27.6±1.57 | 29.6±4.6 | 27.8±3.02 | 23.4±0.6 | 30.4±4.5 |
| **BASDAI, mean ± SE** | 2.58±1.20 | 2.34±1.66 | 3.92±0.70** | 0.69±0.29 | 0.51±0.26 |
| **Disease activity (AAU)** | 0 | 5 (100%) | 4 (80%) | 0 | 0 |
| **Disease Activity (AS)** | 4 (80%) | 0 | 4 (80%) | 0 | 0 |
| **HLA-B27 Positive** | 5 (100%) | 5 (100%) | 5 (100%) | 5 (100%) | 0 (0%) |
| **Biologic/DMARDs** |  |  |  |  |  |
| **Yes** | 4 (80%) |  | 3 (60%) |  |  |
| **No** |  | 5 (100%) |  | 4 (80%) | 5 (100%) |
| **Unknown** | 1 (20%) |  | 2 (40%) | 1 (20%) |  |

^†^ P-values were calculated using the t-test by comparing with HLA-B27 negative HC.

**Supplementary Table 2: HLA-B27 allele subtype status for the study cohort**

| **Study Subject** | **Condition** | **HLA-B27** | **HLA-B(1) Subtype** | **HLA-B(2) Subtype** |
| --- | --- | --- | --- | --- |
| SC01 | AS | Positive | B*27:02:01:01 | B*44:02:01:01 |
| SC02 | AS | Positive | B*27:05:02:01 | B*27:05:02:01 |
| SC03 | AS | Positive | B*27:05:02:01 | B*40:01:02:01 |
| SC04 | AS | Positive | B*27:05 | B*51:ND |
| SC05 | AS | Positive | B*27:05:02:01 | B*40:01:02:01 |
| SC06 | AS+ AAU | Positive | B*27:05:02:01 | B*35:03:01:01 |
| SC07 | AS+ AAU | Positive | B*27 | NA |
| SC08 | AS+ AAU | Positive | B*27:04:01 | B*51:01:01:01 |
| SC09 | AS+ AAU | Positive | B*27:05 | B*35:20 |
| SC10 | AS+ AAU | Positive | B*27:05:02:01 | B*51:01:01:01 |
| SC11 | AAU | Positive | B*62:ND | B*27:ND |
| SC12 | AAU | Positive | B*27:05:02:01 | B*40:02:01:01 |
| SC13 | AAU | Positive | B*27:05 | B*35:ND |
| SC14 | AAU | Positive | B*27:05:02:01 | B*35:01:01:01 |
| SC15 | AAU | Positive | B*27:05:02:01 | B*44:02:01:01 |
| SC16 | HC | Positive | B*27:05:02:01 | B*44:02:01:01 |
| SC17 | HC | Positive | B*15:01:01:01 | B*27:05:02:01 |
| SC18 | HC | Positive | B*27:05:02:01 | B*27:05:02:01 |
| SC19 | HC | Positive | B*15:01:01:01 | B*40:01:02:01 |
| SC20 | HC | Positive | B*27:05:02:01 | B*27:02:01:01 |
| SC21 | HC | Negative | B*07:04:01 | B*40:01:02:01 |
| SC22 | HC | Negative | B*13:02:01:01 | B*57:03:01:01 |
| SC23 | HC | Negative | B*13:02:01:01 | B*44:02:01:01 |
| SC24 | HC | Negative | B*08:01:01:01 | B*08:01:01:01 |
| SC25 | HC | Negative | B*14:01:01:01 | B*40:02:01:01 |

NA: HLA-B27 was tested outside of our lab and HLA-B27 positive information was ascertained from their medical record. The second allele information was not available.

## Supplementary Table 3: Number of QC-passing cells in each condition/contrast.

|  | **TNK cells** | **B cells** | **Myeloid cells** |
| --- | --- | --- | --- |
| **All Subjects** | 245206 | 22893 | 85776 |
| **HCs** | 82233 | 6189 | 26279 |
| **AS** | 41129 | 11269 | 40718 |
| **AAU** | 38232 | 9349 | 40350 |

## Supplementary Table 4: Number of detected (non-zero) genes in each condition.

|  | **Total Cells** | **TNK cells** | **B cells** | **Myeloid cells** |
| --- | --- | --- | --- | --- |
| **Protein coding genes** | 22846 | 20349 | 18923 | 20194 |
| **Long non-coding (lnc) genes** | 18356 | 13300 | 10856 | 12943 |
| **Pseudogenes** | 16572 | 9066 | 6550 | 8528 |
| **Small RNA** | 5097 | 1603 | 1120 | 1528 |
| **Other RNA** | 2427 | 1474 | 967 | 1440 |

**Supplementary Figure 1: Single cell epitope and transcriptome data and comparison with azimuth.** After pseudo-bulking to group individual cells into clusters and quality control (QC) analysis, we that our cell type data collectively clustered as either TNK, B, Myeloid or cells. We subset to each of these major cell types and reprocessed to obtain the clustering maps with unsupervised clustering shown in **A**, **B** and **C**, respectively.

**Supplementary Figure 2: Validation of azimuth cell-types using RNA and protein expression markers.** To confirm the validity of our sub-immunophenotypes, we demonstrate select known markers for each sub-phenotype of TNK, B, and Myeloid cells, such that **A**, **C**, and **E** visualize RNA and **B**, **D**, and **F** show protein markers respectively. Color scales correspond to column-scaled mean library-size-normalized RNA counts (RNA Markers) and column-scaled mean CLR-normalized ADT counts (Surface Protein). Dot sizes correspond to fraction of cells expressing marker genes.

**Supplementary Figure 3:** **Expression Landscape of top DE genes in HC with and without HLA-B27.** Top DE expressed genes per sub-phenotype of each major cell type, are shown in an expression heatmap from 0 to 100, demonstrating the percent of cells in each bin, expressing the genes in rows. Pair-wise hierarchical clustering is done to group similar expression patterns.

**Supplementary Figure 4:** **Expression Landscape of top DE genes in AS subjects and HC with and without HLA-B27.** Top DE expressed genes per sub-phenotype of each major cell type, are shown in an expression heatmap from 0 to 100, demonstrating the percent of cells in each bin, expressing the genes in rows. Pair-wise hierarchical clustering is done to group similar expression patterns.

##

**Supplementary Figure 5:** **Expression Landscape of top DE genes in AAU subjects and HC with and without HLA-B27.** Top DE expressed genes per sub-phenotype of each major cell type, are shown in an expression heatmap from 0 to 100, demonstrating the percent of cells in each bin, expressing the genes in rows. Pair-wise hierarchical clustering is done to group similar expression patterns.

**Supplementary Figure 6: Quantifying the immune landscape in AS+AAU.** AS+AAU subjects were compared with HC with and without HLA-B27 and differential expression analysis was conducted on each of the major cell type lineages of (A) TNK, (C) B and (E) Myeloid cells. Genes with significant (FDR adjusted *p-val*<0.05) differential expression in comparison to HLA-B27^neg^ are shown on the left and comparison with HLA-B27^pos^ HC in shown on the right. Each sub-phenotype are labeled with a unique color in these volcano plots. Cellular enrichment of these sub-phenotype from (B) TNK, (D) B and (F) Myeloid cells were computed as chi-square residuals and significant differences are shown as a heat map with orange and blue depicting enrichment and depletion (respectively) of these cellular sub-populations. The values displayed in the heatmaps reflect *chi-squared* residuals of observed vs. expected frequencies of each cell subset within the indicated conditions. Positive residuals indicate a higher-than-expected frequency (enrichment), and negative residuals indicate a lower-than-expected frequency (depletion). Comparison with all HC as well as AS and AAU only subjects are on the left panel and comparison with HC separated on the basis of HLA-B27 along with AS and AAU subjects is shown on the right panel. Venn diagrams showing the difference in numbers of significantly altered genes between different disease groups are shown for (G) TNK cells, (H) B cells, and (I) myeloid cells.

**Supplementary Figure 7:** **Expression Landscape of top DE genes in AAU subjects and HC with and without HLA-B27.** Top DE expressed genes per sub-phenotype of each major cell type, are shown in an expression heatmap from 0 to 100, demonstrating the percent of cells in each bin, expressing the genes in rows. Pair-wise hierarchical clustering is done to group similar expression patterns.

## Supplementary Figure 8: SDA component (SDA 65) pDC-monocyte differential signature (PMDS) identifies inflammatory pathways in both AS and AAU. This component has a unique gene signature that distinguishes mainly DCs (positive) from Monocytes (negative). Thus, the positive direction, enriched in AS and AAU, is associated with an active immune response, cytoskeletal organization, and stress management. High scores indicate cells that are actively presenting antigens, responding to immune signals, and maintaining structural integrity. This is typical of dendritic cells (DCs) and CD16+ monocytes, which are crucial in initiating and sustaining immune responses. The presence of these cells in AS and AAU suggests ongoing inflammation and immune activation. Similarly, the negative cells, enriched in HC, reflects a state focused on metabolic processes, mitochondrial function, and transcriptional regulation. High scores indicate cells that are maintaining homeostasis, managing oxidative phosphorylation, and regulating gene expression. This is typical of CD14+ monocytes, which are more involved in phagocytosis, metabolic regulation, and less immediate immune activation. This component's gene loadings are shown in A, sorted by mapping location on the Human chromosome. The top-weighted genes in each direction are shown. The top-loaded genes are used for GO enrichment (see methods), and the results are visualized in B, highlighting potential significant matches. The distribution for the cells are scored by this component is shown split by the (C) immune sub-phenotypes and (D) the conditions. To quantify the differences in these distributions, we computed enrichment assessment of cells that scored either positive or negative in (E) metabolic perturbation in both AS and AAU.

## Supplementary Figure 9: SDA component (SDA 27) TNK Cytotoxic Module (TCM) differentiates disease development in AS and AAU This component has a unique gene signature T and NK cell Cytotoxic Effectors (positive) from Quiescent naive and memory TNK cells (negative). This component's gene loadings are shown in (A), sorted by mapping location on the human chromosome. The top-weighted genes in each direction are shown. The top-loaded genes are used for GO enrichment (see methods), and the results are visualized in (B), highlighting potential significant matches. The distribution for how the cells are scored by this component is shown split by the (C) immune sub-phenotypes and (D) conditions. To quantify the differences in these distributions, we computed enrichment assessment of cells that scored either positive or negative in (E) in both AS and AAU subjects and HC.
