# Supplementary figures and images for: Divergent B-cell and cytotoxic TNK cell activation signatures in HLA-B27-associated ankylosing spondylitis and acute anterior uveitis
Source: Front Immunol. 2025 Mar 7;16:1546429. doi: 10.3389/fimmu.2025.1546429 (PMC11926545; doi:10.3389/fimmu.2025.1546429)

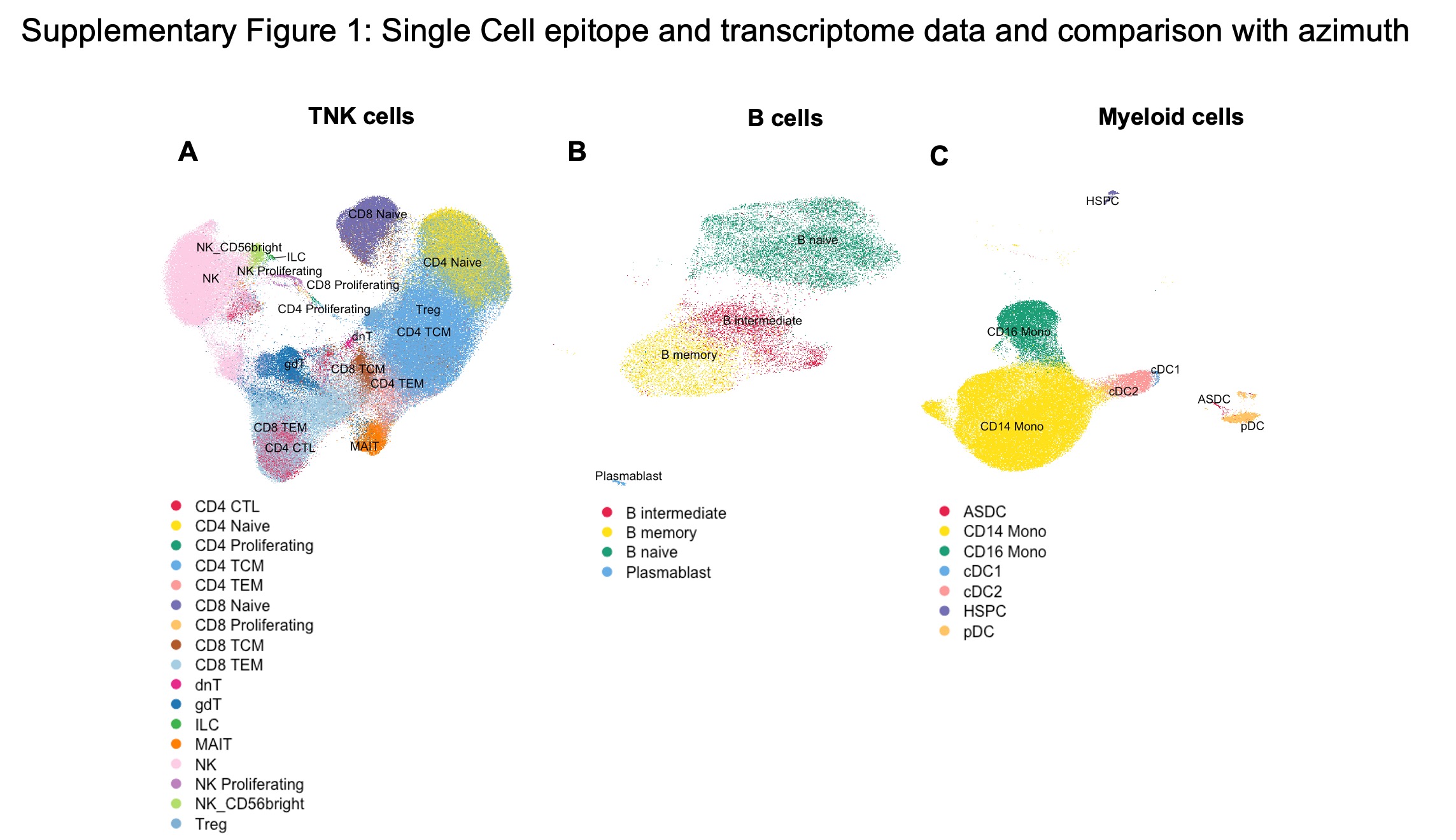

Supplement: Supplementary file 4 [file Image1.jpeg]

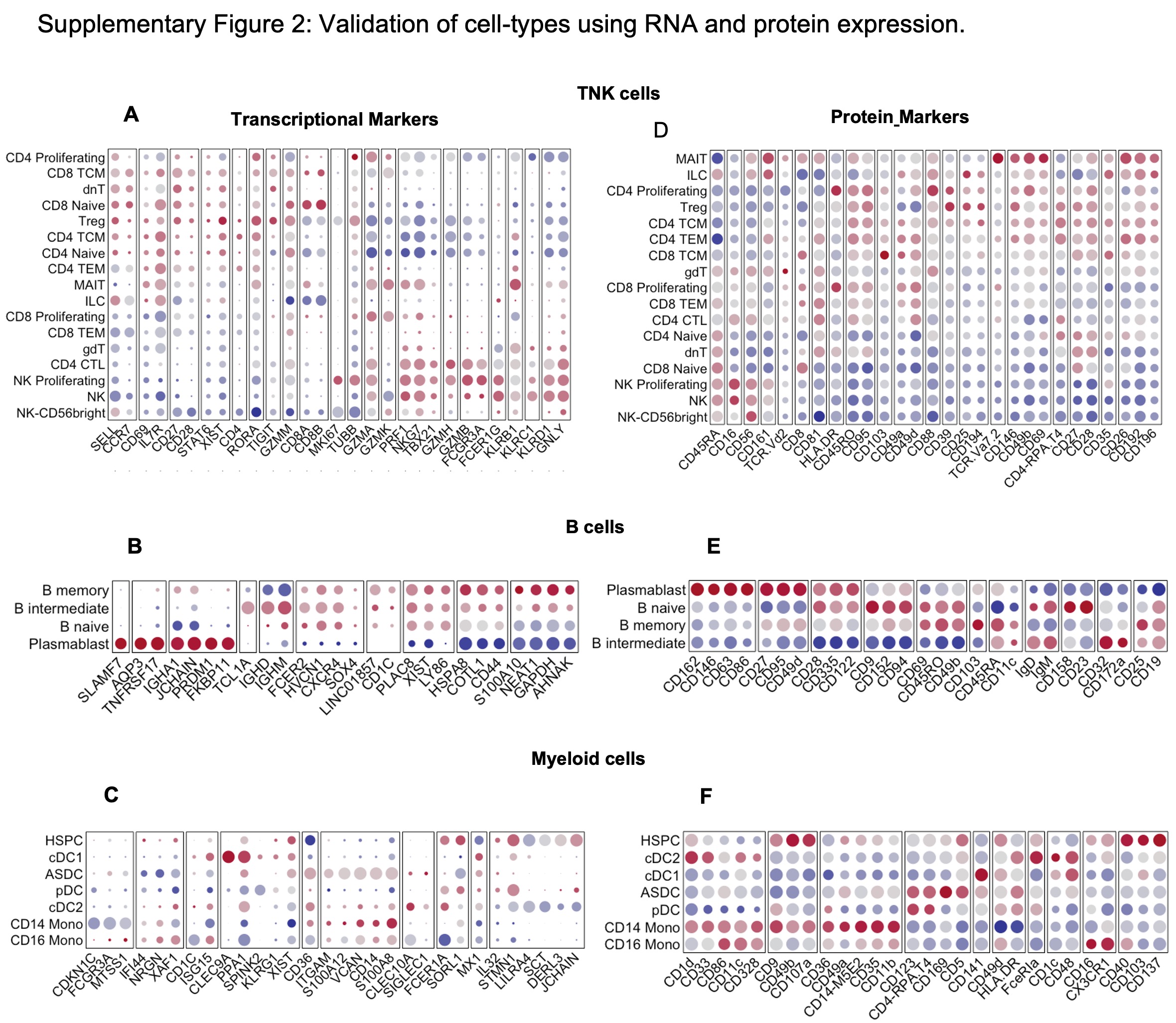

Supplement: Supplementary file 5 [file Image2.jpeg]

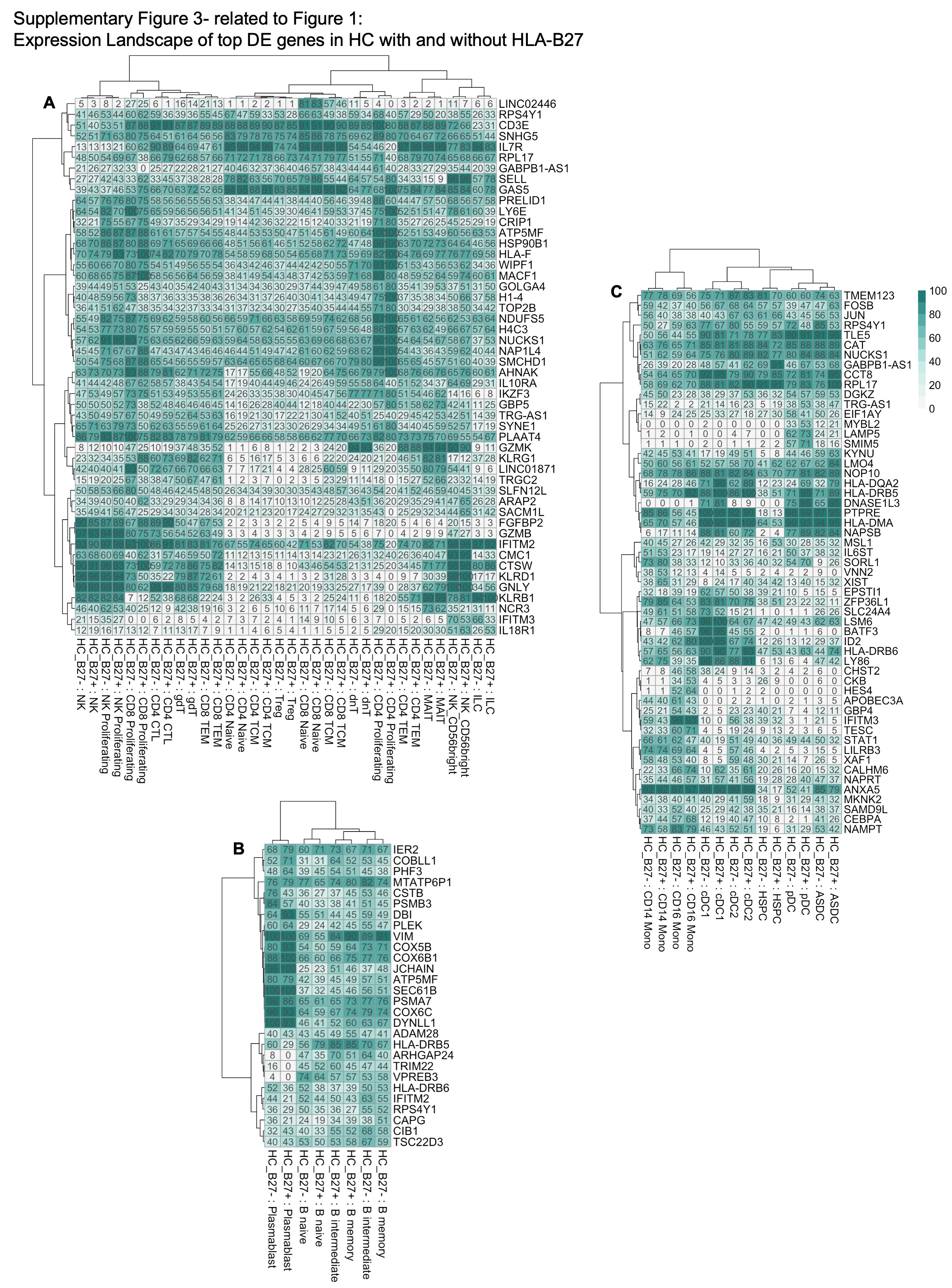

Supplement: Supplementary file 6 [file Image3.jpeg]

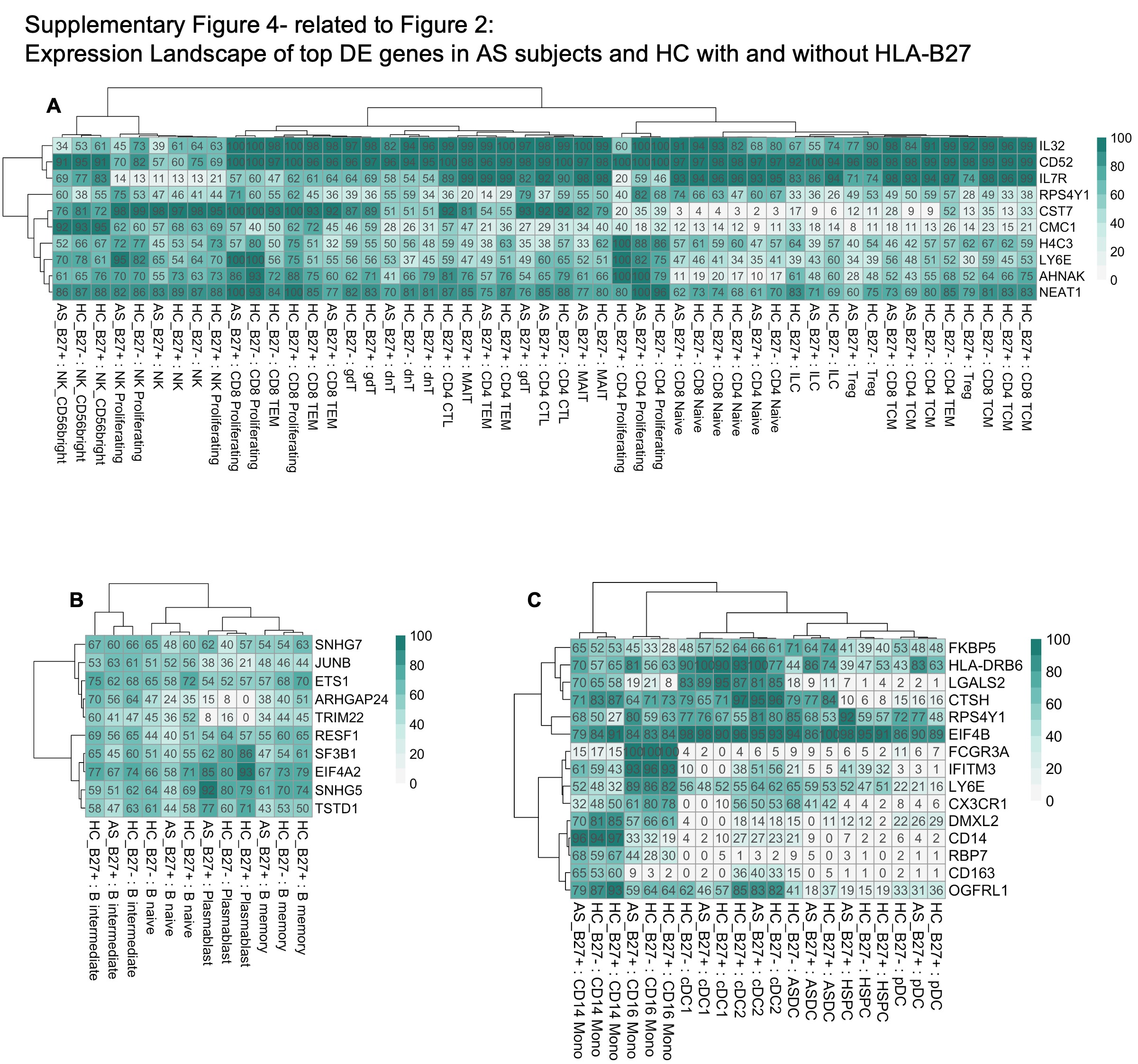

Supplement: Supplementary file 7 [file Image4.jpeg]

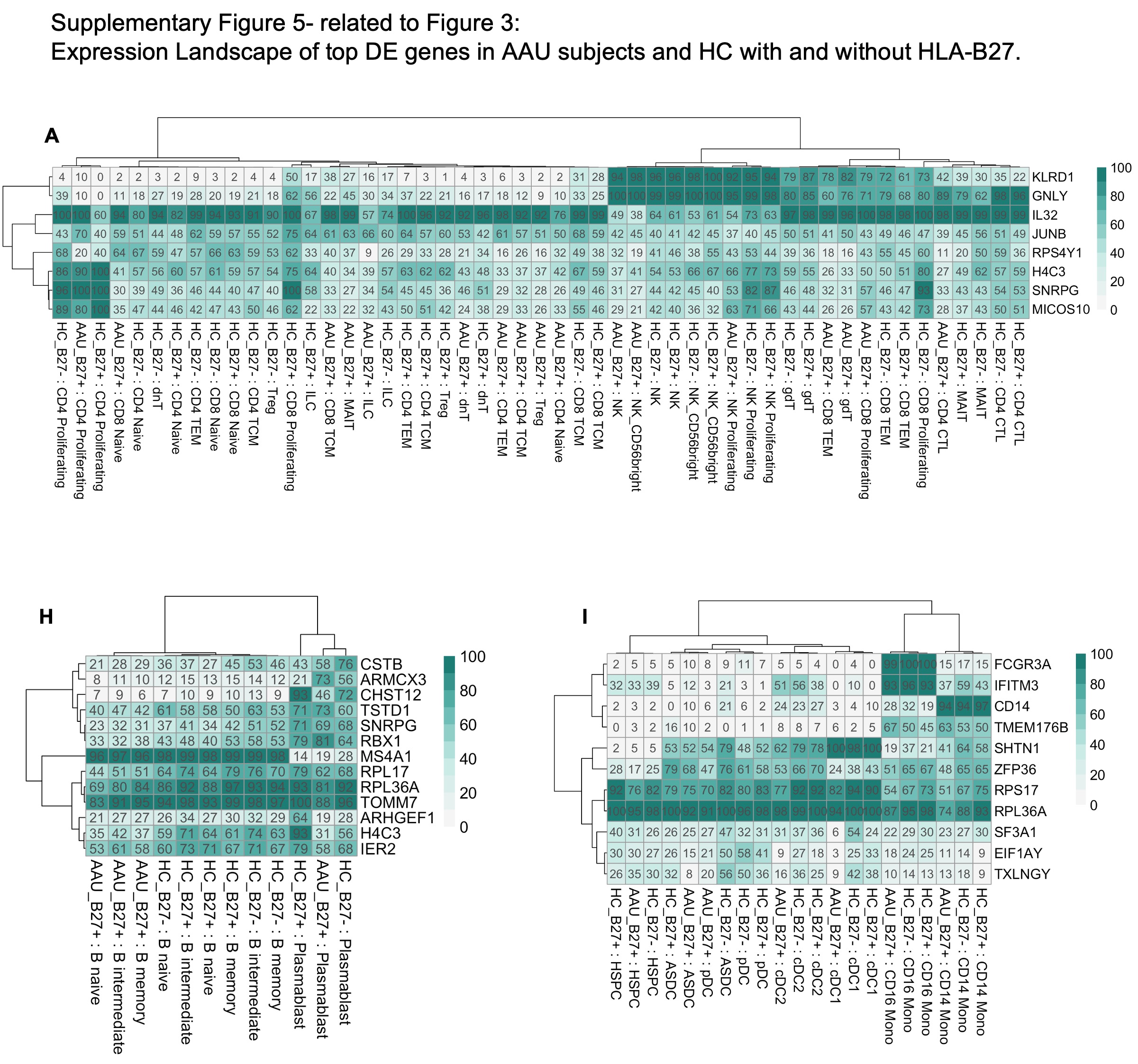

Supplement: Supplementary file 8 [file Image5.jpeg]

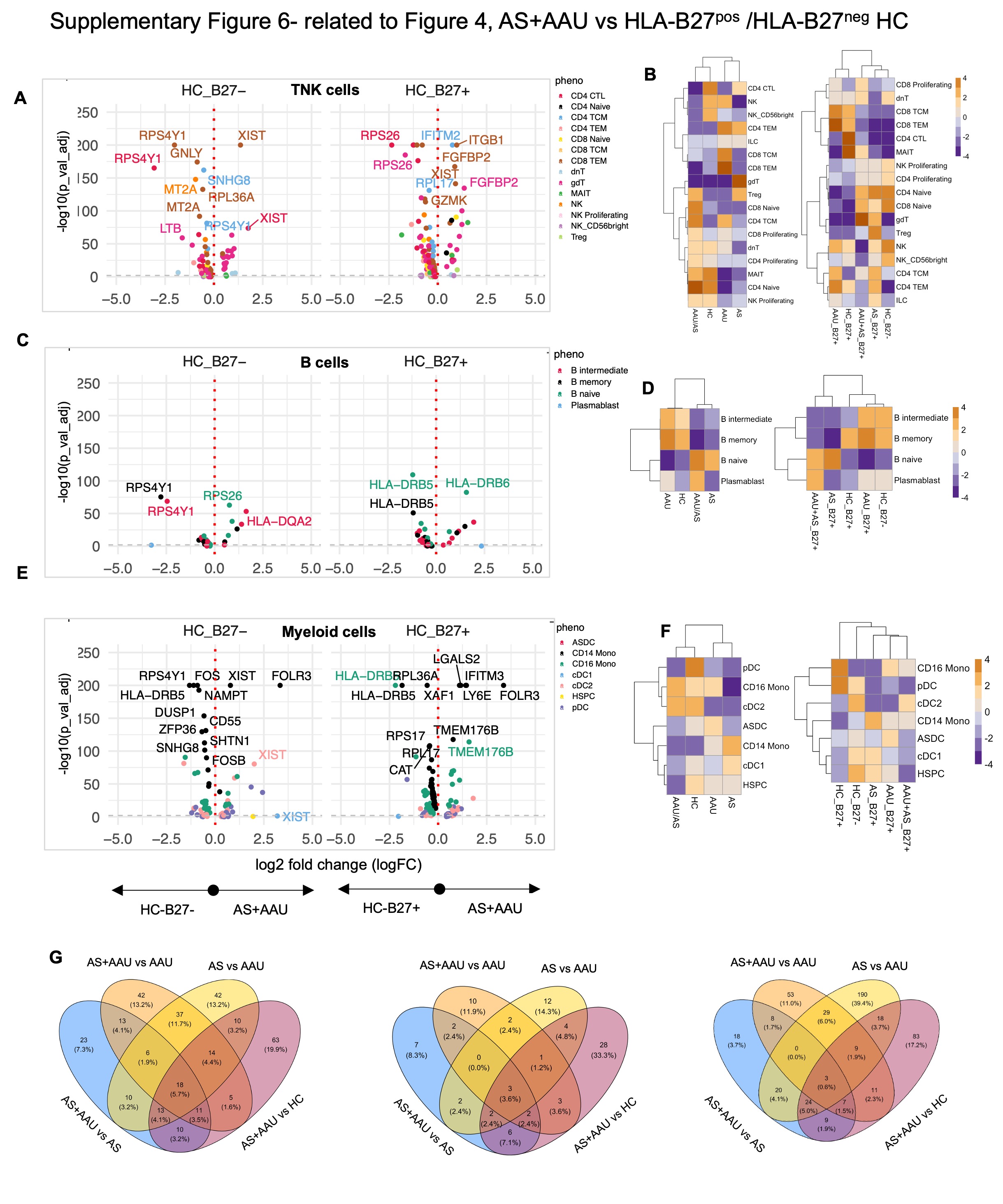

Supplement: Supplementary file 9 [file Image6.jpeg]

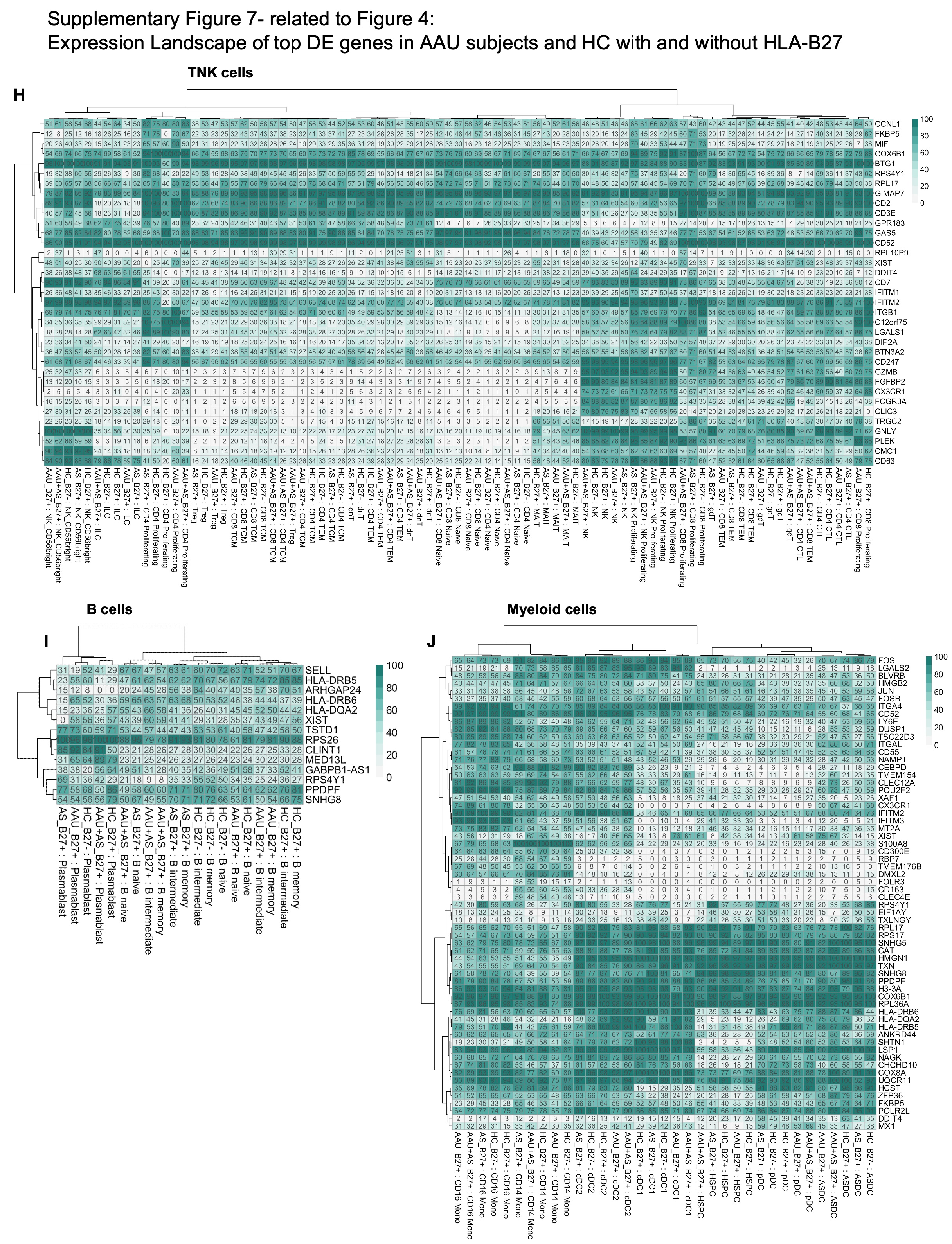

Supplement: Supplementary file 10 [file Image7.jpg]

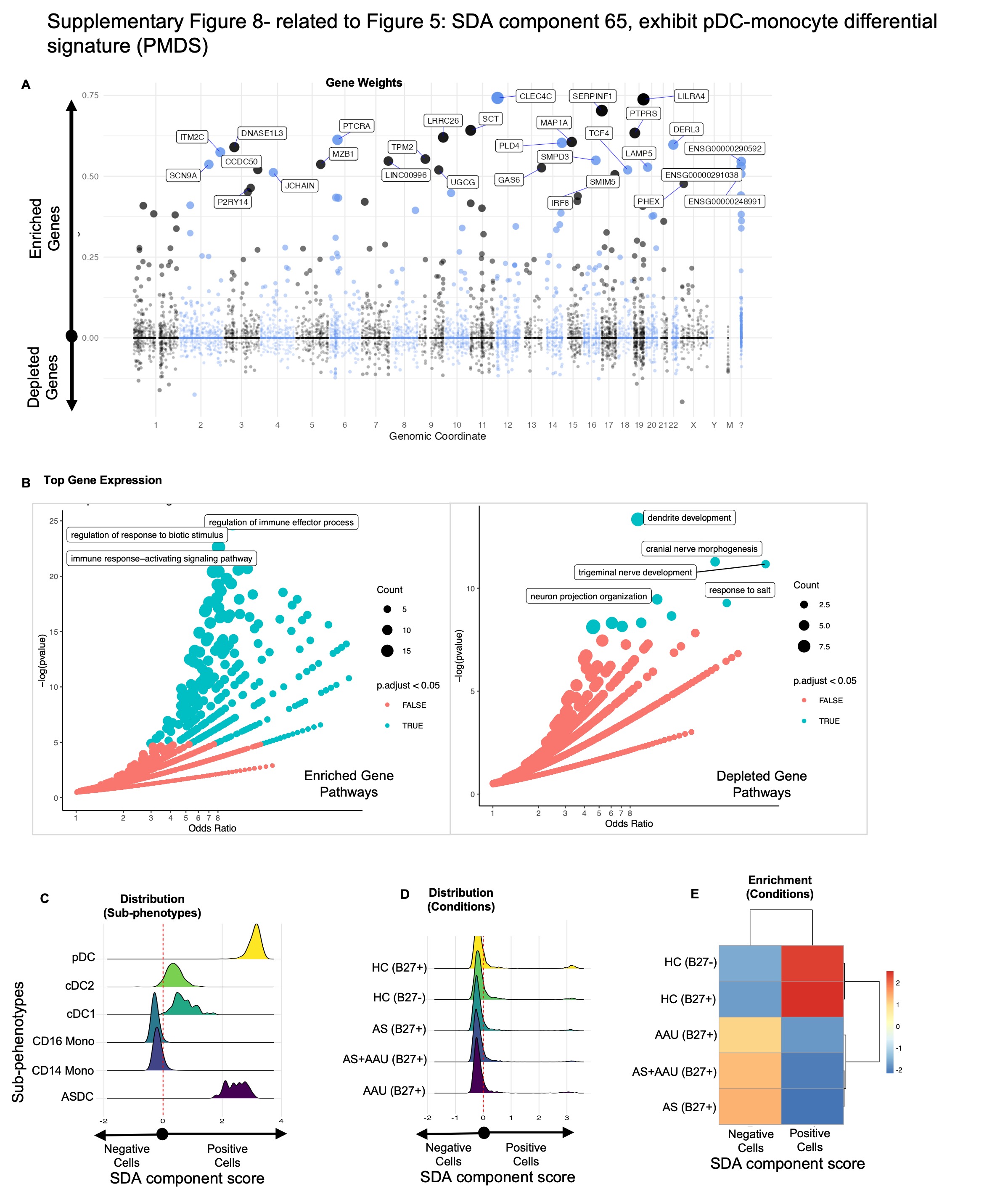

Supplement: Supplementary file 11 [file Image8.jpeg]

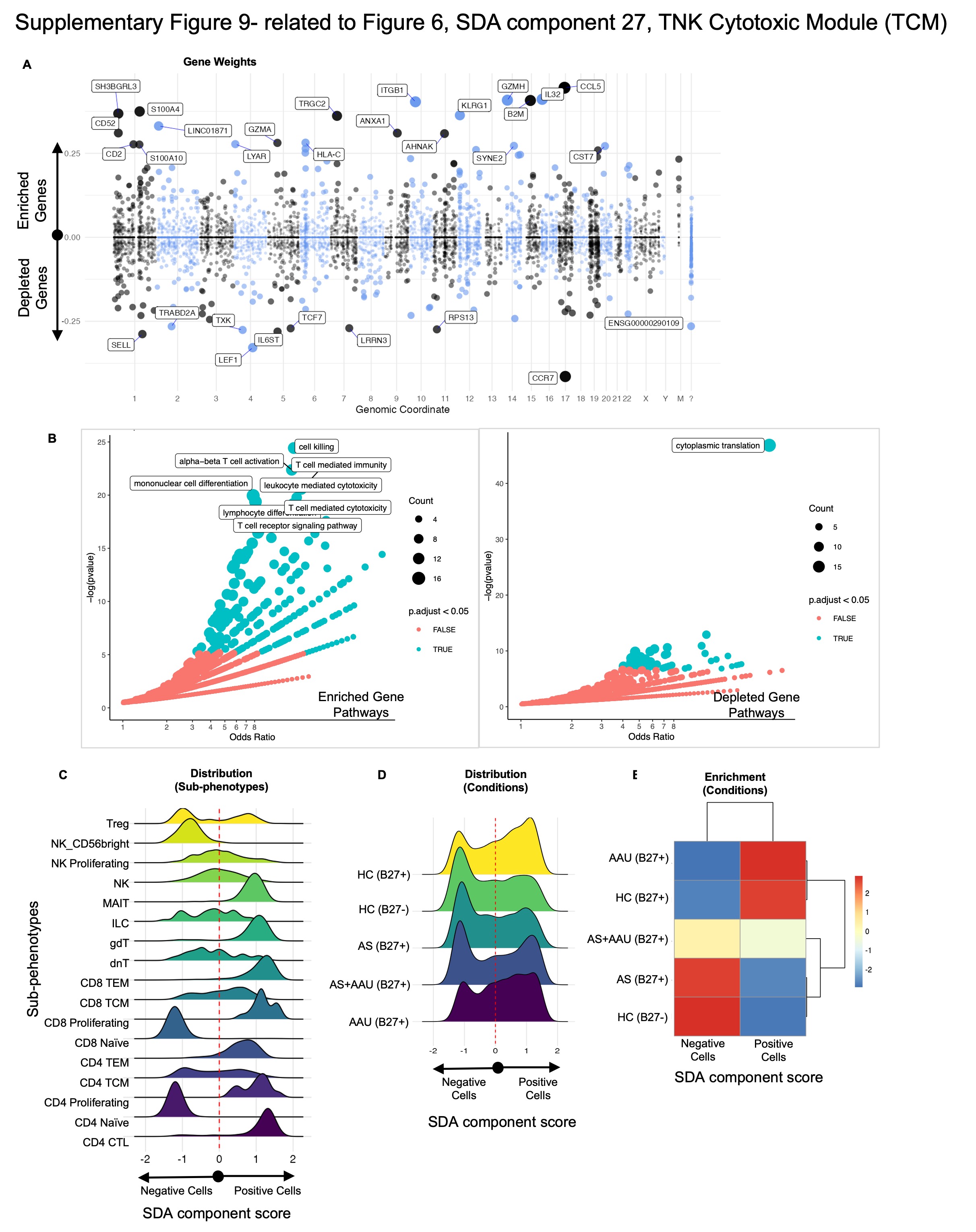

Supplement: Supplementary file 12 [file Image9.jpeg]
